# Crown Plasticity and Competition for Canopy Space: A New Spatially Implicit Model Parameterized for 250 North American Tree Species

**DOI:** 10.1371/journal.pone.0000870

**Published:** 2007-09-12

**Authors:** Drew W. Purves, Jeremy W. Lichstein, Stephen W. Pacala

**Affiliations:** 1 Department of Ecology and Evolutionary Biology, Princeton University, Princeton, New Jersey, United States of America; 2 External Research Office, Microsoft Research, Cambridge, United Kingdom; University of Sheffield, United Kingdom

## Abstract

**Background:**

Canopy structure, which can be defined as the sum of the sizes, shapes and relative placements of the tree crowns in a forest stand, is central to all aspects of forest ecology. But there is no accepted method for deriving canopy structure from the sizes, species and biomechanical properties of the individual trees in a stand. Any such method must capture the fact that trees are highly plastic in their growth, forming tessellating crown shapes that fill all or most of the canopy space.

**Methodology/Principal Findings:**

We introduce a new, simple and rapidly-implemented model–the Ideal Tree Distribution, ITD–with tree form (height allometry and crown shape), growth plasticity, and space-filling, at its core. The ITD predicts the canopy status (in or out of canopy), crown depth, and total and exposed crown area of the trees in a stand, given their species, sizes and potential crown shapes. We use maximum likelihood methods, in conjunction with data from over 100,000 trees taken from forests across the coterminous US, to estimate ITD model parameters for 250 North American tree species. With only two free parameters per species–one aggregate parameter to describe crown shape, and one parameter to set the so-called depth bias–the model captures between-species patterns in average canopy status, crown radius, and crown depth, and within-species means of these metrics vs stem diameter. The model also predicts much of the variation in these metrics for a tree of a given species and size, resulting solely from deterministic responses to variation in stand structure.

**Conclusions/Significance:**

This new model, with parameters for US tree species, opens up new possibilities for understanding and modeling forest dynamics at local and regional scales, and may provide a new way to interpret remote sensing data of forest canopies, including LIDAR and aerial photography.

## Introduction

Forest canopy structure–which can be defined as the sum of the sizes, shapes and relative placements of the tree crowns–is central to all aspects of forest ecology and dynamics. On the one hand, the canopy structure sets the light environment experienced by individual trees, which is known to be a primary determinant of their growth, mortality and fecundity ([1], [2], [3], [4]). These rates are the determinants of the dynamics of species composition, succession and coexistence ([5], [2]). On the other hand, competition for canopy space drives the growth rates, densities, and size distribution of canopy trees, and hence the dynamics of carbon fluxes, carbon storage, timber yields and self-thinning (e.g. [6] pages 213–258). Importantly, the canopy structure itself is set by the sizes, shapes and positions of the crowns of the individual trees. Therefore, canopy structure both determines, and is determined by, interactions among individual trees, defining a feedback that is central to any detailed understanding of forest dynamics ([6] pages 195–398; [2]). Quite apart from its ecological implications, the canopy is the boundary between the land surface and the atmosphere, and so in vegetated regions its structure determines surface properties such as albedo, canopy stomatal conductance and surface roughness, which affect local and regional climate ([7]; [8]; [9], [10]). And a detailed quantitative understanding of canopy structure is required for applications as diverse as estimating regional biogenic VOC emissions ([11]) and remote sensing of forest structure (e.g.[12]).

There is currently no accepted method for scaling from the properties of individual trees to canopy structure. How does the density, size distribution, species, and allometry of the individual trees, determine which trees are in the canopy, the distribution of canopy heights, and the distribution of the total and exposed crown areas? And therefore, how sensitive is canopy structure–and hence forest ecology and dynamics, and forest ecosystem function–to changes in the vital demographic rates of growth, mortality, and recruitment, or to changes in disturbance rates?

The key challenge for any such model is to reconcile the fact that the dimensions and shapes of individual trees are highly correlated with size and species (e.g. see [13]), with the fact that trees are extremely plastic and opportunistic in their growth ([14], [15], [16], [17]). Because of this growth plasticity, canopy trees form complex, irregular, tessellating crowns, that usually do not grow into each other, and that tend to fill most or all of the canopy space (e.g. [18]); whilst maintaining marked size-and species-dependent patterns.

Plasticity is acknowledged to be important in forest ecology ([17]) and in plant ecology in general ([19]), but it has been included in spatial modeling of plant communities only rarely ([20], [21], [22], [23]). Previous approaches to simulating growth plasticity in the context of canopy structure range from simple to highly complex. Forest gap models ([5], [2], [24]) have opted for the simplest approach, assuming no growth plasticity at all. Thus, in these models trees adopt a rigid 3D crown shape that depends on the species and size of the individual, but does not respond to neighbors in any way. But this leads to predictions of canopy structure that contradict observations. For example, the model SORTIE ([2], [24]) reproduces the species composition of northeast US old-growth stands quite well, but for the same forests it predicts extensive interdigitation between adjacent crowns, and too much open space in the canopy. Some modifications to the rigid crown model have been made, by allowing species to alter the position of a rigid crown in relation to the stem base in response to neighbors ([21]), or adjusting the shape of a fixed crown area ([25]). These approaches require a high level of model complexity, but despite this they are not sufficient to capture the combination of plastic crown sizes, irregularity, and space-filling that characterizes real canopies.

Distinct from the forest gap models, some models in the forestry literature have included an explicit consideration of growth plasticity in crown size and shape. The simplest of these models (the Shell model:[26], [27]) allows for only one form of growth plasticity: dropping shaded foliage. The inclusion of this behavior makes the Shell model considerably more realistic than models with the rigid crown assumption, especially because it guarantees a perfectly filled canopy. But it also makes the Shell model orders of magnitude more computationally intensive, and therefore impractical for long-term or large-scale modeling (a similar level of computational intensity is required by the methods outlined in [21] and [25]). An alternative class of models simulates growth at the level of the branch, rather than the individual tree (e.g. [20], [28]), but these models are more complex still.

In this paper, we introduce a simple individual-based model of canopy structure–the ideal tree distribution model, ITD–with tree form (height and crown shape), growth plasticity, and space-filling, at its core. The model is based around assumptions of opportunistic growth and optimal foraging, hence the reference to the ideal free distribution IFD ([29]). Taking a limit of perfect plasticity makes the ITD spatially implicit, meaning that although it is derived from a consideration of spatial processes, it can be implemented without any information on the spatial locations of individuals. This means that the ITD can be implemented extremely rapidly, allowing for parameter estimation using inversion methods, as is done here. We use measurements of crown size and shape from over 100,000 individual trees to parameterize the model for 250 North American tree species. We compare the predictions of the fitted model to the data, and find that the model captures the key patterns of inter- and intraspecific variation in crown size and shape exhibited in the various forest types of the region.

The analysis presented here is the first of a group utilizing the ITD model. Strigul *et al.* (in review) gives the theoretical foundation for the ITD, showing how it can be derived from the Shell model ([26], [27]); explaining mathematically how it leads to a set of so-called macroscopic equations which can be derived explicitly from the properties of trees of different species, to the dynamics of stands; and solved analytically for equilibrium and select transient behaviours. An additional theory paper (Adams *et al.* in review) uses the macroscopic equations from Strigul *et al.* (in review) to explore the dynamics of species invasion, giving the conditions necessary for coexistence, founder control, species dominance, and neutrality, in terms of the life history and biomechanical parameters of the competing species. Purves *et al.* (unpublished) uses a large forest inventory database for the Lake States of the eastern US to estimate these parameters for different species on different soils, showing that the macroscopic equations, and the analytical results in Adams *et al.* (in review), can give accurate predictions for the 100-year dynamics of biomass, size distribution and species composition, and their dependencies on soil. The fact that these results were made possible by the ITD is further evidence of the importance of canopy structure to forest dynamics.

## Methods

### Definition of canopy structure

The canopy structure of a stand of trees can be defined as the sum of the sizes, shapes and spatial arrangement of the individual crowns. These sizes, shapes, and positions, and hence canopy structure itself, are often highly complex, and are likely to depend on many additional details not considered here. We focus on three readily-observed features of an individual tree's crown with immediate functional significance. First, a tree crown is either in the canopy (i.e. at least some of the crown has no other tree's branches above it) or in the understory (i.e., it can only receive light that has passed through the crown of another tree). We refer to this division as canopy status (in or out of the canopy implying canopy status = 1 or 0 respectively). Second, each crown has a projection area (hereafter crown area), defined as the area of ground lying directly underneath the crown (hereafter, we work with crown radius, defined as the radius of a circle with the same area as the crown area). Some or all of this crown area is exposed (exposed crown area, ECA). Third, each crown has a crown depth, defined as the vertical distance between the top of the crown and the lowest living foliage. For the purposes of the analysis presented here, we consider the canopy structure of a stand *q* to consist of the canopy status for every tree in *q*, together with the crown areas and crown depths of those trees in the canopy. The aim of this analysis is to find a simple model that predicts these metrics for each tree in a stand, given the size and species identity of all trees in the stand.

### Model description

Our canopy model, which we refer to as the ideal tree distribution model, is described in detail in Appendix S1. The central assumptions behind the ITD are that (1) the total of the exposed crown areas of the canopy trees in a stand, is logically constrained to be less than or equal to the ground area; (2) if trees are sufficiently plastic in their growth, there should also be no unused canopy space, such that the total of the exposed crown areas is *exactly* equal to the ground area; (3) competition for canopy space is fundamentally height-structured, such that for any stand at any time, there is a critical *canopy height Z* ^*^ such that any foliage above *Z* ^*^ is in the canopy, with all other foliage in the understory.

Then, all that is needed is to solve for the value of *Z* ^* ^that makes condition (2) true. Given the value of *Z* ^*^, the canopy status of an individual tree *i* is set: if *i* is taller than *Z* ^*^ it has some exposed foliage, it which it case it can is classified as a canopy tree (canopy status = 1: see above). Otherwise *i* has no exposed foliage, and can be classified as an understory tree (canopy status = 0). For canopy trees, the crown radius and crown depth are given by trimming the potential crown (see below) at *Z* ^*+^ *V_bias,j_* where *V_bias,j_* a *depth bias* specific to species *j* (see below).

The ITD is most easily understood for the special case where the trees have perfectly flat-topped, disc-like crowns, and no depth bias. In this case, all that is necessary is to sum the potential crown areas of the tallest tree, then the next tallest tree, and so on, until this sum equals the ground area. The height of the last tree added to the sum is *Z* ^*^. All trees included in the sum up to this point (i.e., all trees at least as tall as *Z* ^*^) are assigned to the canopy and given a total, and exposed crown area, equal to their potential crown area; whereas the remaining trees (i.e., trees shorter than *Z* ^*^) are assigned to the understory. This ‘flat top’ version of the ITD retains the key feature of height-structured, density-dependent competition for canopy space, and yet is simple enough to be open to mathematical analysis solving for (for example) the equilibrium *Z* ^*^ in monocultures, and the identity of the late-successional dominant species (Strigul *et al.* in review; Adams *et al.* in review; Purves *et al.* unpublished). Moreover, it gives accurate predictions of canopy status in the tropical forest BCI (Bohlman unpublished). However, here we consider the general version of the ITD, where the crowns can take an arbitrary shape and species can exhibit a depth bias. This version performs better than the flat top version in predicting canopy status and crown dimensions in eastern US forests (comparison not shown), albeit it at the cost of analytical tractability in the context of a dynamic model.

To understand the general version of the ITD, assume that each tree has a potential crown, defined as the shell of foliage that it would adopt if there were no competition with neighboring trees. The potential crown for tree *i* is a function *R_i_*(*Z*), which describes the crown projection radius of a tree's crown at height *Z* above the ground, as a function of the species and size of the tree. This is simply the radius of the circular shadow that the canopy would project, with the sun directly overhead, on to a flat surface held at height *Z* above the ground. For a tree with a conical crown shape, this radius would be a linear function of the distance below the top of the crown.

Second, assume that, given the value of the stand canopy height *Z* ^*^, each tree retains only that part of its potential crown that is above *Z* ^*+^ *V_bias,j_*, dropping all remaining foliage, where *V_bias,j_* is a species-specific depth bias. Under these assumptions, each individual responds to its *effective canopy height Z* ^*+^ *V_bias,j_*, but *Z* ^*^ retains two absolute definitions: *Z* ^*^ is the height that any tree must exceed to be classed as a canopy tree, and *Z* ^*^ is the height that any foliage must exceed to count as exposed. Under this scheme, any proposed value of *Z* ^*^ implies a value for the sum of the exposed crown areas of all of the trees in the stand. This sum decreases monotonically with increasing *Z* ^*^, guaranteeing a unique value of *Z* ^*^ that satisfies the condition that this sum is equal to the ground area (see above). The value of *Z* ^*^ then sets the canopy status, total and exposed crown area, and crown depth, of each tree.

**Figure 1:**
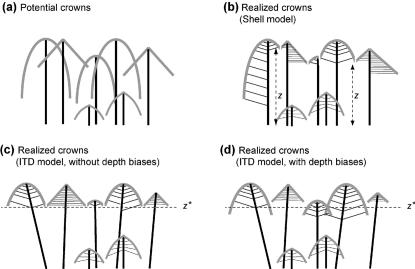
The ITD model, which is spatially implicit, can be derived from the Shell model (a, b), a spatially explicit model that predicts canopy structure from the size and species of individual trees in a stand ([26], [27], and see Strigul *et al.* in review). In the Shell model trees drop shaded foliage, which trims the potential crowns (a) to produce a set of tessellating realized crowns with variable join heights *Z* (b). Under this scheme some trees are predicted to have no crown area, corresponding to understory trees that must be dealt with separately (two smallest trees in figure). The ITD model assumes that, through additional growth plasticity (e.g. angled trunks, as shown) all join heights *Z* become equal to a constant value *Z* ^*^ (c). The version of the ITD model presented in this paper includes a species-specific depth bias *V_bias,j_* such that trees of species *j* trim their potential crowns at an effective join height *Zˆ* = *Z* ^*^+*V_bias,j_*, illustrated in (d). With or without this bias, the value of *Z* ^*^ can be found rapidly, requiring orders of magnitude fewer calculations than the spatially explicit Shell model. In (d) two canopy trees are drawn overlapping in the understory. The ITD makes the assumption that growth plasticity is sufficient to avoid any such overlap, but it is spatially implicit and so does not specify how this would be achieved in a given stand.

Finally, it should be noted here that the ITD can be formally derived from the Shell model (see Strigul *et al.* in review). This is illustrated in Figure 1. In common with the ITD, the Shell model assumes that each tree has a potential crown, and that trees retain only that part that is not shaded by other trees. However, in the Shell model, trees are given no flexibility in the horizontal placement of their crowns, or the foliage within the crowns. This means that, in effect, the canopy height *Z* varies across space, such that a tree of a given species and size would be in the canopy if it was in one location, but in the understory if it was in another location; and that canopy trees can adopt irregular crowns, as they experience different canopy heights *Z* with different neighbours (Fig. 1a). The different values of *Z* must be calculated on a grid of locations, with a resolution fine enough to capture the variation in the size and shape of individual tree crowns (each parcel of space must be assigned to an individual tree). Therefore the Shell model cannot be used with data that lacks the spatial locations of trees (such as the FHM data used here). And even where locations are available, the model is computationally demanding to implement, making it unsuitable for the kind of inverse parameterization used here.

But, with the inclusion of additional growth plasticity into the Shell model, the spatial variation in *Z* approaches zero. Taking a limiting assumption of perfect plasticity then leads to ITD, which assumes a single join height *Z* ^*^ for all locations in the stand.

### FHM data

We parameterized and tested the ITD model against field measurements of canopy status, crown radius, and crown depth, taken from over 100,000 trees as part of the USDA Forest Health Monitoring (FHM) inventory (see [30], [31]). The FHM data covers the coterminous US. The data available at time of download (November 2004) included two time periods (6 years up to and including 1999, and 3 years post-1999). We extracted 6675 plots, containing records for 147,995 living trees, from the pre-1999 data; and 2353 plots, with 63,702 trees, from the post-1999 data. We excluded plantation plots, and plots that were listed as overlapping more than one stand. Within the FHM plots, data were collected from 4 or more separate sampling points. Trees larger than 5 inches dbh (i.e. 12.7 cm) were sampled from a radius of 7.32 m around each point, with a radius of 2.1 m for trees 1–5 inches.

For each tree, a number of observations were provided in the FHM, including species, stem diameter (dbh), crown class, and crown ratio (see below for definitions of these measures). The pre-1999 data provided, for trees larger than 5 inches (i.e. 12.7 cm), two measures of crown projection diameter, but no observed height data. For these data we generated a height value from a height-dbh allometry (Appendix S2). The post-1999 data provided observed heights for all trees, but no crown diameter measurements.

Observed canopy status *U_i_^(obs)^* ( = 1 if tree *i* has any foliage in the canopy, and 0 otherwise) was generated from crown class, which measures the position of a tree in the canopy. We set *U_i_^(obs)^* = 0 where crown class was 5 (‘overtopped’) and to 1 otherwise. For each tree with crown diameter information (i.e. trees over 5 inches dbh in the pre-1999 data), we calculated a value for observed crown radius *R_i_^(obs)^*(m) from *D^(obs)^_i_*_,1_ (m) and *D^(obs)^_i_*_,2_ (m)∶ *D^(obs)^_i_*_,1_ referring to the largest available crown diameter; *D^(obs)^_i_*_,2_ referring to the diameter measured at 90° to D*^(obs)^_i_*_,1_. We calculated observed crown area *α_i_^(obs)^* (m^2^) by assuming an elliptical shape:
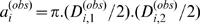
(1)and calculated *R_i_^(obs)^* (m) as 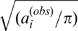. Thus, *R_i_^(obs)^* is the radius of a circle with the same area as the ellipse defined by *D^(obs)^_i_*_,1_and *D^(obs)^_i_*_,2_. An observed crown depth *V_i_^(obs)^* was generated for each tree by multiplying the height of the tree (observed, or from an allometry) by the crown ratio recorded in the field (crown ratio is defined as the length from the top of the crown to the lowest foliage, divided by tree height).

### Parameter estimation

We used maximum likelihood methods ([32]) to estimate species-specific parameters for the ITD for 250 North American tree species. At the center of the parameter estimation was a goodness-of-fit criterion (in this case the log-likelihood) consisting of a comparison of model predictions with observations for all available canopy status, crown radius and crown depth data. Parameter estimation consisted of adjusting parameters to maximize this goodness-of-fit, with standard methods available to estimate the uncertainty in each parameter (Appendix S3).

The predictions of the ITD model for a given stand are determined by the height, potential crown shape, and depth bias, of each individual in the stand (Fig. 1). Values of dbh and height were available for each tree in the data (see above). Thus, the parameters to be estimated consisted of those determining crown shape–i.e. the parameters in the function *R_i_*(*Z*) introduced above–and the depth bias parameter *V_bias,j_*. For crown shape, 4 parameters were required to describe the potential crown shape of canopy trees, with 2 more to describe understory crown dimensions (Table 1). In addition to these 7 parameters, we required 3 statistical parameters to describe unaccounted-for variation in the crown metrics (Table 1: Appendix S3). Thus, a straightforward fit for the full parameter set (hereafter the *full fit*) required 10 free parameters for each species, or 2500 free parameters in total.

**Table 1:** Parameters used in the ITD model, and in parameter estimation.

Parameter	
*D*_0,*j*_, *D*_40,*j*_	Maximum potential crown radius (m) of a tree with dbh 0 cm, 40 cm, of tree of species *j*.
*M_j_*	Crown ratio at which the maximum radius is realized.
*B_j_*	Curvature of crown radius vs distance from top of tree (<1 gives convex; = 1 linear; >1 concave).
*R_us_*_,*j*_	Crown radius (m) of understory tree.
*V_us_*_,*j*_	Crown depth (m) of understory tree.
*T_j_*	Trait score (0–1) of species *j* (used in the single axis parameter estimation scheme).
*V_bias,j_*	Distance above *Z* ^*^ (m) of the base of the crown of trees of species *j*.
σ*_j_, ρ_j _,* φ*_j_*	Statistical parameters describing variation in observed canopy status, crown radius, and crown depth, given model predictions.

The large number of parameters, the inclusion of rare species with few data, and the need for parameter interpretation, motivated a search for approaches with fewer free parameters. To this end we developed the *single-axis* scheme, which reduces the interspecific variation in crown shape parameters to variation in a single species-specific trait score *T_j_*, thus requiring only one free parameter per species. The approach is similar to the way a PCA analysis reduces multidimensional variation to a small number of axes. The scheme can be applied to any analysis estimating multiple parameters for multiple species, and can be extended to include multiple trait axes (see Appendix S3). This parameter estimation scheme required only two free parameters per species: *T_j_* to set the potential crown shape and statistical parameters, and the depth bias parameter *V_bias,j_*. For comparison, we also ran parameter estimations assuming a single crown shape for all species, and/or a single value of *V_bias,j_* for all species, and compared the results with the full fit and the single-axis fit using information criteria (Table 2).

**Table 2:** Comparison of nine alternative parameter estimation schemes.

		Crown shape					
		Full fit		Single-axis		One-shape	
	Species-specific	2500	−157869	516	−174885	259	−191063
		320739	344977	350803	355805	382643	385154
*V_bias,j_*	Global	2251	−159991	267	−179104	10	−196224
		324481	346296	358742	361330	392469	392566
	Fixed at 0	2250	−168456	266	−183036	9	−197192
		341410	363215	366704	369283	394401	394488

Crown shape parameters were either estimated separately for each species (*full fit*); reduced to single axis of variation requiring one free parameter per species (*single-axis*); or reduced to a single crown shape for all species (*one-shape*). Depth bias *V_bias,j_* was either estimated separately for each species; set to a single value for all species (*global*); or fixed at 0. The table gives, for each scheme, the number of free parameters (top left), the maximum log-likelihood (top right), and the value of the AIC and BIC information criteria (bottom left bottom right respectively). Numbers are given to the nearest integer. Lower values of AIC and BIC indicate statistically superior models.

### Predicted-observed comparison

Once parameters had been estimated, a prediction for each of the three crown metrics (canopy status, crown radius, crown depth) was provided for each tree *i* in each inventory plot *q*, by implementing the ITD model in combination with the data for that plot, using the estimated parameter values for the crown shape and depth bias parameters (MLE estimates). For each species *j*, we calculated the mean predicted, and mean observed, canopy status across all trees in all plots, and the mean predicted, and mean observed, crown radius and crown depth for canopy trees. In addition, within each species we recorded the slopes from ordinary least squares (OLS) linear regressions of predicted crown radius vs dbh, and observed crown radius vs dbh (canopy trees only in both cases).

## Results

### Alternative parameter estimation schemes

Parameter estimation schemes without species-specific crown shapes, or without species-specific biases, were strongly rejected by the analysis (Table 2). Both information criteria (AIC and BIC) indicated that the ‘best’ statistical model in this case was the full fit, which included 10 free parameters per species. However, we opt to report the parameter estimates generated by using the single-axis scheme for crown shape, in conjunction with species-specific values for *V_bias,j_*, requiring two free parameters per species. This choice was made for three reasons. First, examination of the model predictions showed clearly this option could recover the key interspecific patterns in canopy status, crown size and crown shape observed in the data (see below). Second, many species are rare, having very few data: for example 38 species have fewer than 30 data in total. For these species 10 parameters almost certainly constitutes overfitting, compromising the predictive ability for the rarer species when used in novel situations. Third, one aim of the analysis was to generate a concise set of parameters that might lend themselves to interpretation in the future. However, we recognize that parameter estimates from the full fit scheme may be more appropriate for making predictions for the more common species in novel data sets. Therefore, any readers interested in using the parameter estimates from the full fit scheme are invited to contact the lead author for the values.

### Interspecific variation in crown shape

**Figure 2:**
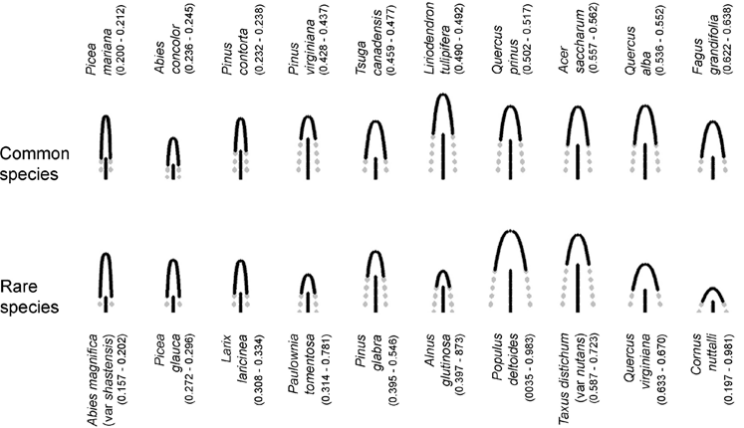
Estimated potential crown shapes for 10 common species and 10 rare species with contrasting estimated crown shapes. Each species is shown at the average height, and average dbh, of that species observed in the data. The full potential crown shape is shown as the grey dashed lines; the solid lines give the realized crown shape drawn at the average observed crown ratio of the species in the data, so the solid lines give a picture of the realized crown shape of a typical individual of the species. Species are given in order of the MLE estimate for the trait score *T_j_* (95% confidence interval for *T_j_* given in parentheses), which is one of the two species-specific parameters estimated, the other being the depth bias *V_bias,j_* (see Appendix S3). *Taxus distichum* (var. *nutans*) is not native to the US, but was found in the inventory data.

Two species-specific parameters were estimated from the data: the trait score *T_j_* and the depth bias *V_bias,j_*. The full set of these two parameters for each of the 250 species in the analysis is given in Tables S1, S2 and S3 including, for each parameter, the MLE estimate and the 95% confidence intervals. Under this scheme, the 6 crown shape parameters, defining the size and shape of the potential crowns of canopy and understory trees, were forced to be perfectly correlated with each other across species, because they were all set by the value of *T_j_* (see Appendix S3). The scheme estimated that the interspecific correlation between the potential crown shape parameters *R*_0,*j*_, *R*_40,*j*_, *R_us_*_,*j*_ and *B_j_* was positive (Table S2). Thus, the scheme estimated that the best single axis of variation between the potential crown shapes of these trees stretches from species with generally narrow, columnar crowns (low *T_j_*), to species with generally wider, more gently curved crowns (high *T_j_*) (see Fig. 2). Pictorial representations of the estimated crown shapes looked reasonable (Fig. 2), including for such unusual species such as Black Spruce (*Picea mariana*) which has a notably narrow, columnar shape (Petrides 1998).

Across all species, *T_j_* varied widely (90% of values within the range 0.14 to 0.86) corresponding to a large variation in crown shape parameters. For example *R*_40,*j*_, which is the maximum potential crown radius of a tree with dbh 40 cm, had a 90% range of 1.72 to 7.96 m. Caution is needed here because many of the species in the analysis were rare, resulting in highly uncertain estimates for species-specific parameters, which will tend to increase the apparent interspecific variation. However, a large range of parameter estimates was seen even in the 30 most common species (e.g. 90% range for *R*_40,*j*_ 2.54 to 5.63 m). The average depth bias *V_bias,j_* was slightly negative (−0.70 m) implying that, on average, species tend to carry a small amount of extra foliage down into the understory (Fig. 1d). But *V_bias,j_* varied very widely across species, with some species exhibiting substantially negative values (90% range −6.47 to+4.90 m for all species; −2.71 to+4.62 m for the 30 most common). A negative value of *V_bias,j_* implies that species *j* carries the bottom of its crown above the canopy height *Z* ^*^.

The interspecific variation in parameters was statistically significant, as indicated by the many pairs of species with non-overlapping credible intervals in either *T_j_* or *V_bias,j_* (Fig. 2 and Table S3: a pair of non-overlapping 95% intervals corresponds to significant difference at *p*<0.0025). This helps to explain why parameter estimates without species-specific crown shapes were rejected (Table 2). The parameter variation in both *T_j_* and *V_bias,j_* was continuous, with no indication of aggregations of species corresponding to distinct functional groups for crown shape or depth bias. There was substantial overlap between the estimated *T_j_* and *V_bias,j_* values of conifer and broadleaf species.

### Predictive ability: interspecific variation

**Figure 3:**
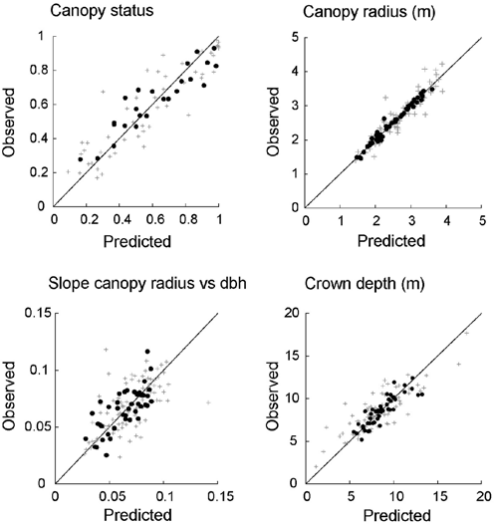
Observed vs predicted average crown metrics for the 75 US tree species with at least 100 measurements of observed canopy status (top left), and the 135 species with at least 30 measurements of observed crown radius and crown depth (remaining panels). The most common third of the species in each panel are shown in black, with remaining species in grey. Predictions are from implementing the ITD model for each FHM forest inventory plot. The parameter estimation allowed two free parameters for each species *j*. Observations for species *j* were generated by averaging all available canopy status data for *j*, and all available crown radius and crown depth measurements for canopy trees of species *j*. Slope refers to slope from a least-squares linear regression of either observed canopy radius vs dbh, or predicted canopy radius vs dbh.

The fitted model reproduced the most important aspects of interspecific variation in crown size and shape (Fig. 3). For species with at least 100 crown class observations (i.e. fraction of trees with some foliage in the canopy), the observed *average canopy status* had a mean of 0.560 (with range 0.169 to 0.976). This compares to a mean model prediction of 0.556, and a mean absolute deviation between the model prediction and observation of 0.096 (i.e., model within 10% of observed for an average species). A similar accuracy could be seen for the other metrics. For species with at least 30 observations of crown radius, the *average crown radius* had an observed mean of 2.65 m (with range of 1.40 to 4.22 m), compared to a mean model prediction of 2.68 m; and the mean deviation between the model prediction and observation was 0.118 m (i.e. predicted crown radius wrong by 12 cm for an average species). The observed *OLS slope of crown radius vs dbh* had an average of 0.0692 m cm^−1^, with range 0.0217 to 0.2198, compared to a mean model prediction of 0.0696; for these slopes, the mean absolute deviation between model and observed was 0.0145 (i.e. 0.01 m radius per cm dbh). The observed *average crown depth* had a mean of 8.630 m (range 2.02 to 18.82), compared to a mean prediction of 8.632; and the average absolute deviation between the model and observed average crown depth was 0.834 m.

### Predictive ability: inter-individual variation

**Figure 4 pone-0000870-g004:**
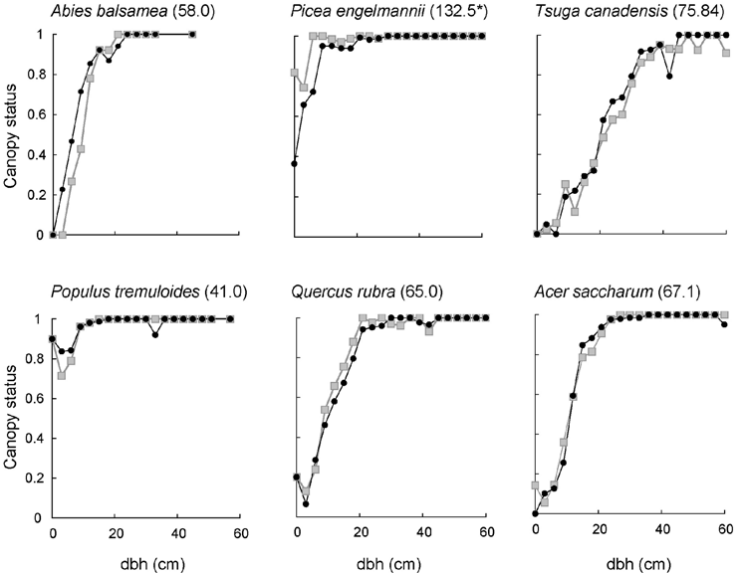
The relationship between dbh and average canopy status for 6 species of contrasting allometry and life history, predicted (grey) and observed (black). The values are the fraction of individuals within the appropriate dbh class that had some foliage in the canopy. Values are only given where there were at least 30 trees in the dbh class for the species. The parameter estimation allowed only two free parameters for each species. Values in parentheses are the successional age of the species, calculated after [50]: high values indicate a late successional species. The successional age of *Picea engelmanni* should not be compared with the other species in the figure because it is the only western species: its successional age is greater than the average for western species, indicating that is late successional.

**Figure 5:**
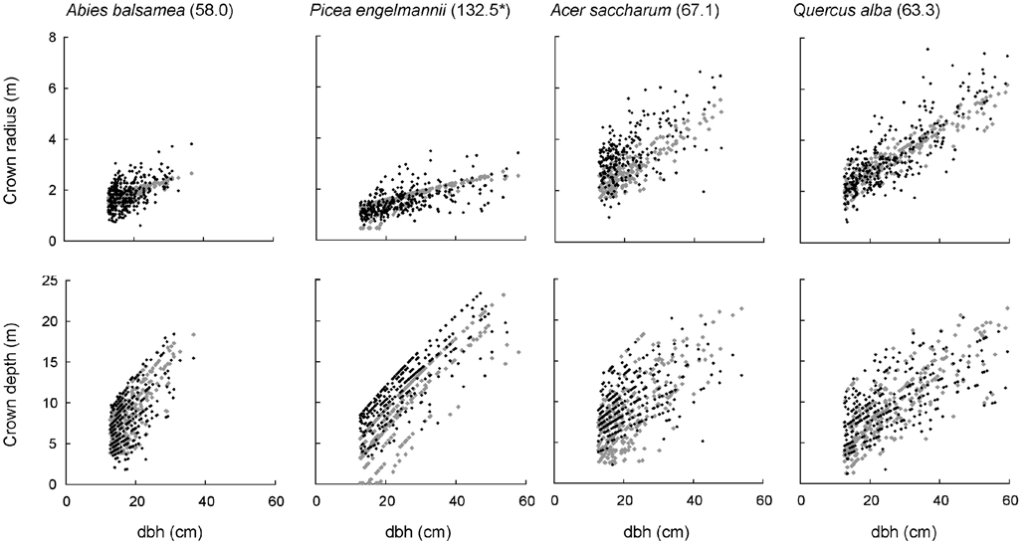
The relationship between dbh and crown radius and crown depth, for 4 common US tree species of contrasting crown shape and allometry: predicted (grey) and observed (black). For each species and metric, data from 300 randomly selected individuals are shown. The parameter estimation allowed two free parameters for each species. Values in parentheses are successional ages: see legend for Fig. 4.

The model also captured the key features of inter-individual variation in crown size and shape within a species (Figs. 4, 5). For all species, larger trees were more likely to be in the canopy, but the slope and curvature of the relationship varied greatly between species (see examples given in Fig. 4). For those species with sufficient data to assess the fit between predictions and observations, the correspondence between model predictions and observations was extremely close (examples given in Fig. 4). This match included non-intuitive results such as the fact that, for many species, the smallest trees were more likely to be in the canopy than were trees of intermediate size (e.g. *Populus tremuloides* and *Quercus rubra* in Fig. 4).

In addition, the model predicted substantial variation in both crown radius and crown depth for a given species and dbh (Fig. 5). Such variation could have been recreated easily by simply implementing the ‘noise’ parameters describing unaccounted-for variation, thereby introducing unexplained random variation to each model prediction; but this was not done here. Instead, the predicted variation was deterministic, resulting solely from the variation in the calculated value of *Z* ^*^ from plot to plot, which itself was driven solely by observed variation in the neighborhoods experienced by trees of a given species and dbh.

For crown depth, the predicted variation was close to that observed, showing that this variation can be explained solely as the deterministic reaction of trees to variation in their neighborhoods. For crown radius, the predicted variation, though substantial, was less than observed. This is likely to reflect some combination of the model missing processes that might affect crown radius (e.g. soil type); and measurement error for crown radius, both of which are included implicitly in the parameters that govern unexplained variation.

## Discussion

### The ITD as a model of canopy structure

The ITD model is evidently able to provide accurate predictions for canopy structure, given data on the individual trees, in a wide variety of forest types, even where only two parameters (*T_j_* and *V_bias,j_*) are allowed to vary between species (Figs 3–5). For a given species, the value of these two parameters, in combination with species-specific height-dbh parameters, predicts average canopy status, crown radius and crown depth (Fig. 3); the within-species relationships between these 3 metrics and dbh (Figs 4, 5); and much of the observed variability for a given dbh (Fig. 5). Thus the ITD model appears to be a relatively parsimonious model, reconciling different species-specific patterns of canopy structure, and crown size and shape, in a wide variety of forest communities, into a simple modeling framework. As such, we hope that it may act as an important step toward a deeper understanding of canopy structure in forests, and possibly in other plant communities.

In evaluating the significance of these results, it is important to bear in mind that describing these patterns with separate allometries would require at least nine species-specific parameters (i.e. for canopy status vs dbh, crown radius vs dbh, and crown depth vs dbh, including the unexplained variation in each), whereas the results presented here used only two free parameters. More importantly, standard allometries predict metrics from dbh alone (e.g. [33], [34] for crown area), and are therefore fundamentally incapable of capturing the fact that the metrics depend on the state of the forest stand within which a tree is found. For example, an allometry of canopy status vs dbh would make a tree of a given dbh equally likely to be in the canopy, whether it was found in an open field, a young stand, or an old growth forest. Allometries can be modified to take into account some measure of stand density (such as basal area), but this requires additional parameters, and the appropriate stand metrics and functional forms are not known. Compared to this, the ITD gives a simple, biologically reasonable formulation that naturally captures competition for canopy space, and that appears to work quite well in a wide range of forest types (Figs 3–5). On the other hand, the conceptual differences between the ITD and the use of standard allometries should not be overstated. Like standard allometries, the ITD assigns a crown shape to each tree–the potential crown shape–which is a simple function of species and size. The advantage of the ITD is simply that it allows for interactions between these potential shapes, to give plasticity in the realized crowns. This then leads naturally to a prediction of which trees are in or out of the canopy.

### Model limitations

The form of the ITD model presented here suffers from a variety of limitations. This includes the lack of time lags in the response of canopy structure to changes in the size and density of trees, such that newly-vacated canopy space becomes filled instantly. In contrast, it is known that tree-fall gaps can take several years to close (e.g. [35]). Also, the potential crown shapes are assumed to be invariant to variation in soils, climate or light environment, whereas they may be variable (e.g. [36]); individuals are assumed to switch instantly between canopy and understory forms, whereas they may show more gradual, and potentially complex, shifts; and the ITD assumes infinite horizontal growth plasticity, whereas trees are obviously highly constrained. These limitations are not dealt with here, because the intention was to provide a simple model that may or may not require modification in the future. In addition, the available observations (the FHM forest inventory data) provided no significant evidence of problems in the model predictions. Therefore, although the current model limitations may prove to be crucial for some applications, their signature is apparently not present in the measurements that are typically taken in forest inventories. As such, their solution appears to require alternative, field-based, observational or experimental approaches. We also note that our analysis did not rule out the possibility that a different model structure could have provided an equally good fit to the data, which further motivates detailed field observations of the key processes determining forest canopy structure.

### Remote sensing of forest structure

A primary goal of the remote sensing of forests is to infer properties of forest stands (the species, sizes and spatial densities of the individual trees, and hence the basal area and biomass of forest stands: hereafter referred to as stand structure), from aircraft-or satellite-borne optical (e.g. [37], [38]) and LIDAR returns (e.g. [39], [40], [41], [42], [43]). The ITD provides a general and rapidly-implemented way to predict these canopy properties from stand structure, and it therefore has the potential to improve the interpretation of remote sensing data. For example, LIDAR returns can provide measurements of canopy height (i.e. the height of the canopy above the ground). In a general sense a taller forest canopy indicates not only taller individual trees, but also trees with larger dbh (because individual height and dbh are positively correlated), along with a lower density of trees (since stand density tends to be negatively correlated with the average size of individuals both within stands of a given species, and among stands of different species: [44]). The ITD can provide quantitative predictions of these relationships for a given forest type. For any given structure (i.e. a list of the species, sizes and densities of individual trees), the ITD predicts not only the maximum canopy height (the height of the tallest tree), and the minimum canopy height (*Z* ^*^), but it also predicts the exact probability distribution of canopy heights. At the heart of the ITD is the function *α_q_* ^(*tot,Zq*)^ which, for a stand *q*, gives the total exposed crown area (ha ha^−1^) vs distance from the ground, *Z_q_* (Appendix S1). This function is exactly equal to the probability of a given LIDAR measurement returning a canopy height greater than *Z_q_* (also see Fig 1d). Therefore the ITD can be used to assess which species, densities and sizes of trees are consistent with a given sample of LIDAR returns. This method would engage with the whole sample of return heights, rather than summary statistics such as the mean height. And the ITD would not require fine-resolution data in order to extract the locations and heights of individual trees; it could be used equally well with LIDAR samples spread coarsely over a wide area. Similarly, analysis of aerial photography data can provide estimates of the density of trees in the canopy, and the probability distribution of their exposed crown areas, ECAs. For any given stand structure, the ITD predicts both of these properties.

In both cases, it should be possible to use a formal probabilistic framework to compare the ITD and the remote sensing data to estimate stand properties from given canopy properties. The ITD does require height allometry and crown shape parameters for each species, and these are, at present, only available for North American trees (Table S3); but these parameters could be derived using forest inventory data from other parts of the world using the methods outlined here.

### Understanding and predicting forest dynamics

The key to understanding, and therefore predicting, the dynamics of mesic forests is the process of density-dependent competition for canopy space, and hence light. This is recognized in forest gap models–the only models that have hitherto been successful in predicting the community dynamics of mixed species forests (e.g. [5], [2]). In these models, complex light-tracing algorithms are used in combination with crown allometries to calculate the degree of shading cast by, and experienced by, different individuals. Growth and mortality are then functions of the level of light incident on each individual. And in forestry it has long been recognized that both crown class (a more general measure of canopy status) and crown ratio are important predictors of the growth and mortality of individual trees.

But modeling the process of height structured competition in a quantitative manner that allows for a rigorous understanding and predictive ability for forest dynamics has remained elusive. In part, this has been due to a lack of data with which to parameterize models of canopy structure, and/or the effects of canopy structure on the growth, mortality and reproduction of individuals. This was especially true for measurements that are hard to take (e.g. exposed crown areas, leaf densities, understory light), for rates that are slow and therefore require large sample sizes (e.g. mortality rates for canopy trees), and for rare species. However, this problem is rapidly being overcome by the appearance of large forest inventory data sets, which contain observations of growth rates, and mortality and reproductive events (e.g. the USDA FIA contains millions of tree records for the US alone: see [11]) and measurements relevant to canopy structure (e.g. the FHM data used here). Satellite-and aerial-photograph derived data sets could be larger still.

A more fundamental limitation has been the lack of a simple quantitative model relating the state of a forest stand (the species, sizes and densities of trees) and canopy structure, and hence competition for canopy space and light. This relationship appeared to be complex and spatially explicit, and hence computationally expensive and mathematically intractable. Forest gap models have utilized computationally intensive light-tracing algorithms to generate predictions for incident light levels experienced by the center of the crown of each individual (e.g. [2]). But even these models have lacked a biologically-derived formulation for competition between the largest canopy trees, which cast little shade on each other, but which compete for canopy space (and yet large trees are responsible for most of the carbon fixation, and hence NPP and carbon storage, of forests). Approaches to understanding competition between large trees in forest gap models have been limited to phenomenological neighbourhood models ([45], [46], [47]). This work has lead to important new discoveries in forest ecology, but neighbourhood models require the estimation of parameters for every *pair* of species (i.e. interaction-specific parameters), and there is no reason to expect them to recover the fundamental fact that stand level NPP is limited by the total available canopy space. In contrast, a class of forest models derived from the Shell model (e.g. TASS, see [48]) is ideally suited to understanding competition between canopy trees, but this is at the cost of an extremely computationally intensive 3D tessellation algorithm.

Being spatially explicit, the ITD can be implemented much more rapidly than the canopy component of either forest gap models, or the Shell model. Moreover it can be used in conjunction with data that is not spatially explicit (e.g. most forest inventory data, including the FHM and FIA). Similarly to the Shell model, the ITD is all about dividing up canopy space and so, in common with it, it naturally captures competition between large trees: growth can simply be a made a function of exposed crown area, ECA ([49]). This allows for realistic competition between large trees whilst constraining stand-level productivity, without the need for interaction-specific parameters. The dynamic model that results from replacing the canopy structure component of a standard forest gap model (SORTIE: [2], [24]) with the ITD, is also rapid to implement, and becomes analytically tractable for such metrics as equilibrium density, basal area and biomass, patterns of self thinning, and the invasion of monocultures by other species (Strigul *et al.* in review). And yet it can reproduce the dynamics of SORTIE, provided that SORTIE is first made more realistic by the inclusion of extra growth plasticity (Strigul *et al.* in review).

Thus, the combination of forest models based on the ITD, with large forest inventory data sets, may represent a first step toward a rigorous understanding and predictive ability for forest dynamics. In recent work we show that this can lead to accurate predictions for the 100-year dynamics of biomass, size distribution and species composition, and their dependency on soil type, for the forests of the Lake States of the eastern US (Purves *et al.* unpublished). Perhaps this approach can be extended to make the model parameters explicit functions of climate and soil, leading to a rigorous, individual-based understanding of observed regional variation in forest structure and species composition. If so, defensible predictions of the nature and timescale of forest responses to climate change and other anthropogenic perturbations could be within reach.

## Supporting Information

Appendix S1 Derivation and description of the ideal tree distribution (ITD) model
(0.34 MB DOC)

Appendix S2 Estimation of species-specific height-dbh parameters
(0.05 MB DOC)

Appendix S3 Parameter estimation scheme
(0.28 MB DOC)

Table S1 Summary of ITD parameter estimates the single axis scheme, with the additional free parameter *V_bias,j_*. Parameters marked with * were fit as species-specific free parameters; for species *j*, parameters marked with {double dagger} depended only on the value of *T_j_*. Parameter *M_j_* was fixed at 0.95 for each species *j*, as shown. By definition, 5% of the species have values for parameter P above the 90% range for P, and 5% have values below the range. This interval was calculated for each parameter, either using only the 30 most common species, or all species, as shown.
(0.06 MB DOC)

Table S2 Parameters for converting the trait score for species *j*, *T_j_*, to crown shape parameters (see eq. S3.3). Parameters marked *fixed* were not estimated, but fixed at the values given. Other parameters were fit as global free parameters, as part of the single-axis scheme. These values can be used with eq. S3.3 to assign species-specific crown shape parameters to species *j*, from the value of the trait score *T_j_* given in Table S3.
(0.04 MB DOC)

Table S3 ITD model parameters for the 250 tree species represented in the FHM data, together with height-dbh parameters for North American tree species not present in the FHM data. The height *H_i_* (m) of a tree of species *j*, with *dbh_i_* in cm (or diameter at root collar, *drc_i_*) can be calculated as *H_i_* = 10ˆ[*a_j,dbh_*+*b_j_*.log_10_ (*dbh_i_*)], or as *H_i_* = 10ˆ[*a_j,drc_*+*b_j_*.log_10_ (*drc_i_*)] . The parameter *T_j_* can be used in conjunction with Table S2 to generate species-specific crown shape parameters for species *j*. *V_bias,j_* is a species-specific parameter required by the ITD model. The numeric code used to identify species in US Forest Service forest inventories is given. 95% confidence intervals are given for *T_j_* and *V_bias,j_* in parentheses.
(0.46 MB DOC)
