# Supplementary material for: Crown Plasticity and Competition for Canopy Space: A New Spatially Implicit Model Parameterized for 250 North American Tree Species
Source: PLoS One. 2007 Sep 12;2(9):e870. doi: 10.1371/journal.pone.0000870 (PMC1964803; doi:10.1371/journal.pone.0000870)
Supplement: Appendix S1 — Derivation and description of the ideal tree distribution (ITD) model (0.34 MB DOC) [file pone.0000870.s001.doc]

DW Purves *et al.,* ‘Crown plasticity and competition for canopy space: a new spatially implicit model parameterised for 250 North American tree species’.

**APPENDIX S1: Derivation and description of the ideal tree distribution (ITD) model**

*Model description.* The ITD model predicts, for each tree in a stand, the canopy status (in or out of the canopy), the total crown area (defined as the area directly underneath the crown), the exposed crown area (the area of the crown receiving light direct from the sky, as viewed directly from above), and the total crown depth (distance from the top of the crown to the base of the crown). These metrics are predicted from the dbh, height and species of each tree in the stand, without requiring any information on the spatial location of individuals. The ITD works by solving for $Z^{*}$, the precise definition of which is the minimum height *z* above the ground above which the total of the exposed crown areas is equal to the ground area. With no depth bias (Fig 1c), $Z^{*}$ is equal to the join height at which adjacent canopy crowns meet. But with depth biases (Fig 1d) the more precise definition given above is required. The value of $Z^{*}$ then sets the canopy status, crown area and crown depth of each tree (see below).

To calculate $Z^{*}$, we begin by defining $a_{i}^{(Z_{q})}$, which is the crown area of tree *i* over $Z_{q}$, where $Z_{q}$ is a proposed value of $Z^{*}$ for plot *q* (a visual explanation of $a_{i}^{(Z_{q})}$ is given in Fig. S1). The sum over all $a_{i}^{(Z_{q})}$ in plot *q* then defines $a_{q}^{(tot,Z_{q})}$, which is (therefore) the total crown area above height $Z_{q}$. So, by definition, $a_{q}^{(tot,Z_{q})}=1$ ha ha^-1^ when $Z_{q}=Z_{q}^{*}$. The value of $a_{i}^{(Z_{q})}$is determined by the crown radius $R_{i}^{(Z_{q})}$ given $Z_{q}$:

$R_{i}^{(Z_{q})}$ = if $H_{i}\geq Z_{q}$

= 0 if $H_{i}<Z_{q}$

$a_{i}^{(Z_{q})}$ = $\pi[R_{i}^{(Z_{q})}]^{2}$ (S1.1)

where $R_{i,\max}^{(p)}$ is defined in eq. S1.1., and the height $\tilde{Z}_{i}$ is related to the *effective canopy height* to which tree *i* responds, $\hat{Z}_{i}$, given its depth bias $V_{bias,j}$, such that $\hat{Z}_{i}=\max(Z_{q}+V_{bias,j},0)$ (see Fig. 1d); the value of $\tilde{Z}_{i}$is then given by .


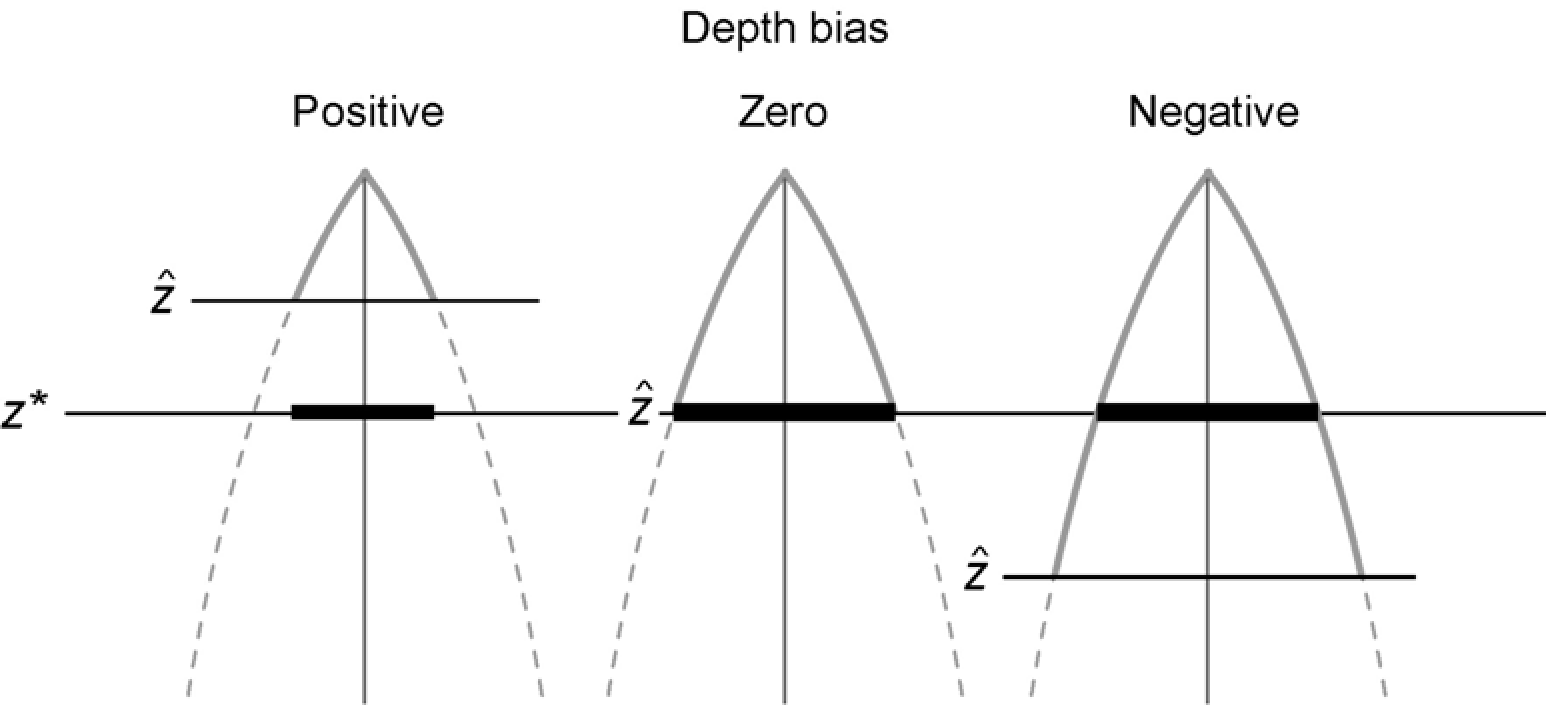


**Fig S1.1.** Definition of $a_{i}^{(Z_{q})}$, $\hat{Z}_{i}$and $\tilde{Z}_{i}$ for species with different depth biases $V_{bias,j}$ but sharing the same $Z^{*}$. The dashed lines gives the potential crown; the solid lines gives the realized crown; and the thick horizontal line is the crown projection at height $Z^{*}$. As the figure shows, for species with positive depth bias (left), the crown projection at $Z^{*}$ is equal to the total crown projection, i.e. the projection at the effective height $\hat{Z}_{i}$. The crown projection at $Z^{*}$ is also equal to the total crown projection at $\hat{Z}_{i}$ where the depth bias is zero, although in this case, by definition, $\hat{Z}_{i}$and $Z^{*}$ are the same (middle). However, where the depth bias is negative (right), the crown projection at $Z^{*}$ is smaller than the crown projection at$\hat{Z}_{i}$. Thus, overall, the crown projection at height $Z^{*}$ is given by the projection at, where $\tilde{Z}_{i}$ is defined as the maximum of $Z^{*}$ and$\hat{Z}_{i}$.

The difference between $\tilde{Z}_{i}$ and $\hat{Z}_{i}$ stems from the fact that trees of species with negative depth biases carry a larger total crown projection area than the area they project at height $Z_{q}$, whereas trees of species with positive depth biases project their full crown projection onto height $Z_{q}$ (Fig. S1). Note that eq. S1.1 follows directly from the formula for crown shape (eqs. S1.6 and S1.7 below).

The total canopy projection area at height $Z_{q}$, $a_{q}^{(tot,Z_{q})}$, is then:

$a_{q}^{(tot,Z_{q})}=\sum_{i\in q} a_{i}^{(Z_{q})}$ (ITD model: individual) (S1.2)

The extension to the cohort ITD approximation, which applies where only the densities of individuals of different sizes and species is known, begins by defining a *cohort i*, which is a collection of identical individuals occurring at a density $w_{i}$ across space (ha^-1^). The total crown projection area at height $Z_{q}$for the cohort, given join height $Z_{q}$, is equal to the crown projection area of any individual in cohort *i*, multiplied by the density $w_{i}$, so:

$a_{q}^{(tot,Z_{q})}=\sum_{i\in q} w_{i}a_{i}^{(Z_{q})}$ (ITD model: cohort) (S1.3)

which has an obvious connection both with eq. S1.2, and with the structure of the FHM data, which uses variable radius sampling (specifically, different plots sizes for trees with dbh larger or smaller than 5 inches) which requires expansion factors $w_{i}$ to reconstruct the average properties of the stands represented by the inventory (Hanson *et al.* 1992). In this data, the value $w_{i}$ is referred to as an expansion factor, and is calculated as

$w_{i}=1/[N_{q}A_{i}]$ (S1.4)

where $A_{i}$ is the subplot area (ha) for trees with size $dbh_{i}$*,* and $N_{q}$ is the number of subplots from which trees were sampled from plot *q*.

In both cases, the ITD proceeds by (1) proposing a value of $Z_{q}^{*}$ for a stand (i.e. plot) *q*; (2) using eqs. S1.1 – S1.3, in conjunction with the current crown shape parameters, to define the total canopy area given $Z_{q}^{*}$, $a_{q}^{(tot,Z_{q})}$; (3) reducing $Z_{q}$ if $a_{q}^{(tot,Z_{q})}<1$ ha ha^-1^and increasing $Z_{q}^{*}$ if $a_{q}^{(tot,Z_{q})}>1$ ha ha^-1^; (4) repeating steps 2-3, adjusting step sizes accordingly, until $Z_{q}^{*}$ is known to within 1 cm (in practice we find that repeating steps 1-3 between 10 and 15 times is usually sufficient to determine $Z_{q}^{*}$ within 1 cm); (5) calculating the predicted realized canopy status $U_{i}^{(pred)}$, crown radius $R_{i}^{(pred)}$ and crown depth $V_{i}^{(pred)}$ for each tree *i* in plot *q* given the value of $Z_{q}^{*}$:

$U_{i}^{(pred)}$ = 0 if $H_{i}<Z_{q}^{*}$

= 1 if $H_{i}\geq Z_{q}^{*}$

$R_{i}^{(pred)}$ =

$R_{i}^{(pred, exposed)}$ =

$V_{i}^{(pred)}$ = $\min(\max(H_{i}-\hat{Z}_{i}^{*},0),H_{i})$ (S1.5)

where the values of $\hat{Z}_{i}^{*}$and $\tilde{Z}_{i}^{*}$ are as above, evaluated at $Z_{q}=Z_{q}^{*}$; and $R_{i}^{(pred, exposed)}$ is the exposed crown radius, defined as the radius of a circle with the same area as the exposed crown area (ECA: i.e.$ECA_{i}=\pi[R_{i}^{(pred, exposed)}]^{2}$). ECA was not utilised in this analysis, because it was not measured in the FHM inventory: we include the formulation for ECA here because it is an important model prediction for use in other settings (see discussion).

Finally, it should be noted that in some plots, $Z_{q}^{*}$ has no solution as defined above. This occurs where $a_{q}^{(tot,Z_{q})}$ is less than 1 when$Z_{q}=0$, which corresponds to plots where the total of the maximum potential crown areas is not sufficient to fill the available space. In this case, we set $Z_{q}^{*}=0$. This situation corresponds to stands where some bare ground is exposed to the sky, i.e. a canopy with gaps.

*Formula for crown shape.* We adopt a simple 4-parameter formulation to describe the potential crown shape of a canopy tree *i*, from species *j*, given diameter at breast height $dbh_{i}$ (cm). First, we define the maximum potential crown radius $R_{\max,i}^{(p)}$ as

$R_{\max,i}^{(p)}= R_{0,j}+[R_{40,j}-R_{0,j} ].[dbh_{i}/40]$ (S1.6)

where the parameters $R_{0,j}$ and $R_{40,j}$ (m) are, for species *j*, the maximum potential crown radius of a tree with dbh 0 and 40 cm respectively . Equation S1.6 was selected to be linear from inspection of plots of observed crown radius vs dbh: the top edge of the cloud of points, which corresponds to individuals expressing the full potential crown, was approximately linear in all cases.

The maximum radius $R_{\max,i}^{(p)}$ occurs at a distance below the top of the crown where the crown ratio is equal to the parameter $M_{j}$ (crown ratio is distance from the top of the tree crown to the base of the crown, divided by the tree height); $M_{j}$is taken to depend on species but not on dbh. Therefore,$R_{\max,i}^{(p)}$ occurs at distance $H_{i}M_{j}$ (m) from the top of the tree, which is at height $H_{i}[1-M_{j}]$ from the ground, where $H_{i}$ denotes the height of tree *i.* Note that the parameter $M_{j}$ is not the maximum crown ratio of species *j*: trees are allowed to adopt any crown ratio, but increasing the ratio beyond $M_{j}$ leads to no further increases in potential crown radius.

The potential crown radius at an arbitrary distance *y* from the top of the tree, $R_{i,y}^{(p)}$, is given as a power function:

$R_{i,y}^{(p)}=R_{\max,i}^{(p)}.\left(\frac{\min(y,H_{i}M_{j} )}{H_{i}M_{j}}\right)^{B_{j}}$ (S1.7)

where the parameter $B_{j}$ sets the curvature of the crown between the top of the tree and the edge of the potential crown: $B_{j}=1$ gives a conical crown; $B_{j}<1$ gives a convex crown; and $B_{j}>1$ gives a concave crown. Note that evaluating eq. S1.7 at $y=0$ gives $R_{i,y}^{(p)}=0$; and $y\geq H_{i}M_{j}$ gives $R_{i,y}^{(p)}=R_{i,\max}^{(p)}$.

For any tree *i*, eqs. S1.6 – S1.7 give a width $R_{i,y}^{(p)}$ for any distance *y* from the top of the tree’s crown, which is associated with a height $H_{i}-y$ from the ground. The functional forms used here were chosen to be rapid to calculate, simple to interpret, and to respond smoothly in response to parameter alteration.

Understory trees, defined as those with height below $Z^{*}$ (see below), are assumed to adopt a species-specific fixed understory radius $R_{us,j}$ and fixed understory crown depth $\min(V_{us,j},H_{i})$. These values are needed to calculate the likelihood (see below), but are not the focus of the study, which aims to understand canopy status for all trees, and the crown area and crown depth of canopy trees only (methods). As such, we urge caution in interpreting and using the parameters $R_{us,j}$ and $V_{us,j}$.

**Appendix S1 Reference**

Hanson MH, Frieswyck T, Glover JF, Kelly JF (1992) *USDA Forest Service General Technical Reports*. **NC – 151**.
