# Supplementary material for: Crown Plasticity and Competition for Canopy Space: A New Spatially Implicit Model Parameterized for 250 North American Tree Species
Source: PLoS One. 2007 Sep 12;2(9):e870. doi: 10.1371/journal.pone.0000870 (PMC1964803; doi:10.1371/journal.pone.0000870)
Supplement: Appendix S2 — Estimation of species-specific height-dbh parameters (0.05 MB DOC) [file pone.0000870.s002.doc]

**Appendix S2: Estimation of species-specific height-dbh parameters**

Data used to estimate height-diameter allometries were downloaded from the USDA Forest Service *Forest Inventory Analysis* (FIA) website (<http://www.fia.fs.fed.us/>). The FIA inventory closely related to the FHM, but it is much larger, and does not include observations of crown radius. We used all data that were available from the FIA at time of download (March 2005). We estimated allometries for 339 species, which includes all species listed in the current (March, 2005) and older versions (ca. 2000) of FIA documentation. Height-dbh parameters for each of these species are given in Table S3. We used a hierarchical modeling approach that allowed us to estimate parameters for species with little or no data (see below).

For some species, many more stems were available than were needed for analysis. Therefore, we used the following algorithm to sample from the available data: If ≤ 45 stems were available for a species, we used all of them. If > 45 stems were available, we sought to create a sample that contained 15 stems in each combination of the following categories: three diameter categories (lower, middle, and upper third of the diameter distribution within each species) × three physiographic categories (stems on plots classified as xeric, mesic, and hydric) × two crown class categories (canopy and understory; ‘canopy’ refers to the FIA classes open grown, dominant, and co-dominant; ‘understory’ refers to the FIA classes intermediate and overtopped). If ≤ 15 stems were available in a category, we used all of them. If > 15 stems were available in a category, we accepted each stem with probability 15/*n*, where *n* is the number of available stems in the category. This algorithm yielded an expected maximum of 270 stems per species. Some states in the FIA reported few heights for saplings (diameter 2.5-12.7 cm), so that for many species, the algorithm described above produced samples containing few saplings. Therefore, for each species, if fewer than a third of the total stems sampled by the above algorithm were saplings, we sampled additional saplings (if any were unused and available) to obtain a maximum sapling proportion of one third of the stems per species. If more saplings were available than necessary to obtain a one third sapling proportion, we took a random sample of the available saplings. The data-set that we analyzed had ≥ 1 stem for 281 of the 339 species, > 30 stems for 216 species, and 36,878 total stems.

Visual inspection of the data suggested that diameter-height relationships were linear, or nearly linear, for all species in log-log space. Therefore, for each species, we estimated an allometry of the form log_10_(height) = *a* + *b*×log_10_(diameter). FIA diameter measurements were either diameter at breast height (‘dbh’; where breast height is 1.37 m) or diameter at root collar (‘drc’). Root collar measurements were primarily for dry woodland species in the western U.S. with multiple stems. A few species had both dbh and drc data, so we defined separate intercepts (parameter *a*) for dbh and for drc stems within each species as follows:

$h_{ij}=a_{j,dbh}\lambda_{ij}+a_{j,drc}(1-\lambda_{ij})+b_{j}d_{ij}+\varepsilon_{i}$, (S2.1)

where *h_ij_* is log_10_ of height(m) of stem *i* in species *j*; *a_j_*,*_dbh_* is the intercept for species *j* dbh stems; *a_j_*,*_drc_* is the intercept for species *j* drc stems; λ*_ij_* is one for dbh stems and zero for drc stems; *b_j_* is the slope for species *j*; *d_ij_* is log10(diameter); and $\varepsilon_{i}\sim\text{normal}(0,\sigma^{2})$, where *σ*^2^ was assumed to be constant across species. The hierarchical model we implemented assumes individuals nested within species, which are nested within genera, which are nested within one of three groups (*Pinus*, other conifers, and angiosperms), which are nested within an overall ‘root.’ The likelihood of the data-set is

$\begin{matrix} L=\prod_{i}^{Nstems} P\left[h_{i}|d_{i},\lambda_{i},\sigma^{2},SP(i)\right]\text{ }\times\prod_{j}^{Nspecies} P\left[SP_{j}|GEN(j),\sigma_{sp}^{2}\right]\text{ }\times\\ \text{ }\prod_{k}^{Ngenera} P\left[GEN_{k}|GR(k),\sigma_{gen}^{2}\right]\text{ }\times\prod_{l}^{Ngroups} P\left[GR_{l}|ROOT,\sigma_{gr}^{2}\right]\text{ }, \end{matrix}$ (S2.2)

where all probabilities are normal densities; *h_i_* is log_10_(height) of stem *i*; *d_i_* is log_10_(diameter) of stem *i*; λ*_i_* is one if the diameter of stem *i* was measured at breast height and zero if measured at the root collar; *σ*^2^ is the variance of the error in *h_i_* among individuals within species; ***SP***(*i*) and ***SP****_j_* are, respectively, vectors of parameters (*a_dbh_*, *a_drc_*, and *b*) for the species of stem *i* and for species *j*, and $\sigma_{sp}^{2}$is a vector (shared by all species) giving the variance among species within genera for the three parameters; ***GEN***(*j*) and ***GEN****_k_* are, respectively, vectors of parameters (*a_dbh_*, *a_drc_*, and *b*) for the genus of species *j* and for genus *k*, and $\sigma_{gen}^{2}$is a vector (shared by all genera) giving the variance among genera within groups for the three parameters; ***GR***(*k*) and ***GR****_l_* are, respectively, vectors of parameters (*a_dbh_*, *a_drc_*, and *b*) for the group of genus *k* and for group *l*, and $\sigma_{gr}^{2}$is a vector (shared by all groups) giving the variance among groups within the overall root for the three parameters; and ***ROOT*** is a vector of parameters (*a_dbh_*, *a_drc_*, and *b*) for the overall root of the hierarchy.

We used WinBUGS software (Spiegelhalter et al. 2004) to generate Bayesian posterior samples (Gilks et al. 1996) for each parameter, using uninformative priors for all parameters. We used normal priors (with mean zero and variance 1000) for the intercepts and slopes, and gamma priors (with mean one and variance 1000) for the inverse of the variances. For our model, this choice of priors allows for Gibbs sampling from the posterior distributions (Spiegelhalter et al. 1996), which is computationally efficient. We used the species-level posterior means as allometric parameter values. For species with abundant data, the posterior means are nearly the same as the maximum likelihood estimates for that species analyzed in isolation. In contrast, for species with no data, the posterior distributions are determined completely by the hierarchical structure of the model. For example, the posterior means for a species with no data in a genus with abundant data will be similar to the posterior means for that genus.

**Appendix S2 references**

Gilks, W. R., S. Richardson, and D. J. Spiegelhalter. (1996). Introducing Markov chain Monte Carlo. Pages 1-19 in W. R. Gilks, S. Richardson, and D. J. Spiegelhalter, editors. *Markov chain Monte Carlo in practice*. Chapman and Hall/CRC, Boca

Spiegelhalter, D. J., N. G. Best, W. R. Gilks, and H. Inskip. (1996). Hepatitis B: a case study in MCMC methods. Pages 21-43 in W. R. Gilks, S. Richardson, and D. J. Spiegelhalter, editors. *Markov chain Monte Carlo in practice*. Chapman and Hall/CRC, Boca Raton.

Spiegelhalter, D., A. Thomas, N. Best, and D. Lunn. (2004). WinBUGS 1.4.1. http://www.mrc-bsu.cam.ac.uk/bugs.
