# Supplementary material for: Crown Plasticity and Competition for Canopy Space: A New Spatially Implicit Model Parameterized for 250 North American Tree Species
Source: PLoS One. 2007 Sep 12;2(9):e870. doi: 10.1371/journal.pone.0000870 (PMC1964803; doi:10.1371/journal.pone.0000870)
Supplement: Appendix S3 — Parameter estimation scheme (0.28 MB DOC) [file pone.0000870.s003.doc]

**Appendix S3: Parameter estimation scheme**

The maximum likelihood analysis aims to find the best fit (MLE) values, and confidence intervals, for the ITD model parameters (denoted $\theta$) given the inventory data (denoted Z). See Hilborn & Mangel 1997. The vector of model parameters $\theta$ is defined as $\theta=\{\theta(1),\theta(2)...\theta(250)\}$ for the set of 250 species, where . Note that $\theta$ can be thought of as consisting of model parameters ($R_{0,j},R_{40,j},R_{us,j},M_{j},B_{j},V_{us,j},V_{bias,j}$), together with additional statistical parameters that describe the variation not accounted for by the model ($\sigma_{j},\varphi_{j},\rho_{j}$: see below). However, these parameters are treated equally by the analysis.

The analysis begins by defining the likelihood function, which quantifies$l(Z|\theta)$, the log-likelihood of the data $Z$, conditional on the model constraints and a given vector of species parameters $\theta$ :

$\begin{matrix} l(Z|\theta)=\sum_{i\in R_{1}} \ln\left\{G(U_{i}^{(obs)}|Z_{q(i)}^{*}, \sigma_{j})\right\}+ \\ \sum_{i\in R_{2}} \ln\left\{N(R_{i}^{(obs)}|R_{i}^{(pred)}, \rho_{j})\right\}+ \\ \sum_{i\in R_{3}} \ln\left\{N(V_{i}^{(obs)}|V_{i}^{(pred)}, \varphi_{j})\right\} \end{matrix}$ (S3.1)

where $N(R_{i}^{(obs)}|R_{i}^{(pred)}, \rho_{j} )$ is the probability density for the observed crown radius$R_{i}^{(obs)}$ (m) given a normal distribution with a mean equal to the predicted realized crown radius $R_{i}^{(pred)}$, and standard deviation $\rho(j)$; similarly $N(V_{i}^{(obs)}|V_{i}^{(pred)}, \varphi_{j} )$ is the probability density for the observed crown depth$V_{i}^{(obs)}$ (m) given a normal distribution with a mean equal to the predicted crown depth $V_{i}^{(pred)}$, and standard deviation $\varphi_{j}$; and $G(U_{i}^{(obs)}|Z_{q}^{*},\sigma_{j} )$ is the probability of observing canopy status $U_{i}^{(obs)}$ given the predicted canopy join height for plot *q*, which we defined as:

$\begin{matrix} G(U_{i}^{(obs)}=1|Z_{q}^{*},\sigma_{j} )=1/[1+\exp(-\sigma_{j}.[H_{i}-Z_{q}^{*}])] \\ G(U_{i}^{(obs)}=0|Z_{q}^{*},\sigma_{j})=1-G(U_{i}^{(obs)}=1|Z_{q}^{*},\sigma_{j}) \end{matrix}$ . (S3.2)

Equation S3.2 is a logit functional form, such that the probability that tree *i* is in the canopy tends to 1 as the difference between $H_{i}-Z_{q}^{*}$ becomes highly positive; tends to zero as $H_{i}-Z_{q}^{*}$ becomes highly negative; and is equal to 0.50 when $H_{i}=Z_{q}^{*}$. The steepness of the relationship is set by $\sigma_{j}$. All three of the terms in eq. S3.1 depend on the values of $Z_{q}^{*}$ for each plot, which themselves are defined by the current parameters $\theta$, together with the data Z. Hence the notation $l(Z|\theta)$.

Equation S3.1 includes three sets of forest inventory plots *q*, and all trees *i* within those plots. The set $R_{1}$contains all trees *i* within plots *q* from the post-1999 inventory data, for which a measure of crown class was given, and which were not assigned to set $R_{3}$. The set $R_{2}$contains all trees *i* within plots *q* from the pre-1999 inventory data, for which measures of crown radius were given, which were listed as canopy trees ($U_{i}^{(obs)}$=1), and which were not assigned to set $R_{3}$. The set $R_{3}$contains randomly-chosen canopy trees ($U_{i}^{(obs)}$=1) from either pre- or post-1999 data. One third of the trees were assigned to set $R_{3}$. These set definitions ensured an approximately equal mix of data from all three metrics to contribute to the log-likelihood $l(Z|\theta)$, whilst allowing each tree to feature only once in the calculation of $l(Z|\theta)$.

Given the definition of the likelihood conditional on parameters and data, $l(Z|\theta)$, as given above, the analysis proceeds by finding the vector of parameters$\theta$ that maximizes $l(Z|\theta)$. The parameter values specified by this vector are referred to as the maximum likelihood estimates (MLE) of the parameters. Because the predicted crown metrics depend not only on the model parameters$\theta$, but also on the species and sizes of the other trees in plot *q*, the MLE parameters for different species could not be estimated separately. Thus, we sought the MLE values, and 95% confidence intervals, using a Metropolis-Hastings Monte Carlo Markov Chain simulated annealing scheme.

*Reducing dimensionality.* The full fit procedure outlined above estimated 10 parameters for each of 250 species, giving 2500 free parameters from just over 120,000 data. Overall, this left a reasonable number of data per parameter (c. 480). However, the different species differed hugely in their abundances, such that many species had very few data in total: for these species, 10 free parameters was likely to constitute over-fitting. In addition, a key aim of the paper was to produce a parameter set that leant itself to interpretation, both within the paper (see results and discussion) and in the future. Together, these called for an approach that reduced the dimensionality of the analysis, i.e. reduced the number of free parameters. To this end, we developed a suite of 9 alternative estimation schemes differing in the number of free parameters, one of which was the free fit scheme described above. The alternative schemes consisted of all factorial combinations of (1) the *full fit* for crown shape (where crown shape all parameters except $V_{bias,j}$, i.e. 9 parameters); (2) a reduction to a single axis of variation in crown shapes (the *single-axis* scheme: see below); and (3) a single crown shape shared by all species (the *one-shape* scheme: see below); crossed with (a) a separate $V_{bias,j}$ for each species; (b) a single value of $V_{bias,j}$ shared by all species; (c) $V_{bias,j}$= 0 for all species. Thus, the 9 alternatives consisted of all combinations 1a – 3c given above, where combination 1a corresponds to the full fit scheme outlined above (2500 free parameters in total), and combination 3c corresponds to a single crown shape for all species, and $V_{bias,j}$ = 0 for all species (10 free parameters in total).

For each alternative, we sought the MLE values and confidence intervals for the free parameters, as described above for the full fit scheme. We then assessed the statistical support for these alternatives by comparing two different information criteria: the Aikaike information criterion (AIC) and the Schwarz information criterion (BIC). These criteria are calculated for a given model given the number of free parameters and the fit to the data (as measured by the likelihood). The model with the lowest value is then considered to be the best supported by the data. Both criteria are ‘penalised’ by the inclusion of the extra parameters, such that additional parameters are only supported if they are justified by a sufficient improvement in fit to the data. There is considerable debate and uncertainty about the use of BIC vs the (currently more conventional) AIC (e.g. see Burnham and Anderson 2004; Kuha 2004). Importantly, the choice is not to do with whether the underlying statistical analysis is Frequentist or Bayesian (Burnham and Anderson 2004). Compared to the AIC, the BIC reflects a stronger prior belief that the data are structured primarily by a low-dimensional model, such that, under situations of large amounts of data as encountered here, it tends to select more parsimonious models compared to the AIC, and thus leads to models that can be interpreted more easily (Kuha 2004). The BIC can also been seen as more appropriate where the aim of the analysis is understanding and interpretability, rather than predictive ability in extrapolation (Kuha 2004).

However, in this case both the AIC and BIC chose the most highly paarameterised model (option 1a, with 10 free parameters per species: see table 1). Importantly, both critera also gave lower values for options 2a,b,c than they did for options 3a,b,c (Table 1). Thus, a single crown shape for all species, and a single species-specific depth bias $V_{bias,j}$, are strongly rejected by the analysis, regardless of the choice of information criterion. In the paper, we chose to report the results from the single-axis crown shapes with species-specific $V_{bias,j}$ (i.e. option a2 given above) even though the information criteria did not indicate these as the most parsimonious model. This choice was made for three reasons. First, examination of the model predictions showed clearly that option 2a, despite having only 2 free parameters per species, could recover the key interspecific patterns in canopy status, crown size and crown shape observed in the data (Figs. 3-5). Second, a key aim of the paper was to generate parameters that leant themselves to interpretation, both within the paper (Fig. 6) and in the future: at this stage of understanding, 2 parameters per species is more appropriate as regards this aim. Third, many species are rare, having very few data: for example 38 species have fewer than 30 data in total, and 17 species have fewer data than the 10 free parameters in the most parameterised model. Therefore, it is highly likely that the improved statistical fit of the most parameterised model primarily represents improvements in the common species, with a high degree of ovefitting for the rarer species, which is likely to compromise the predictive ability for the rarer species when used in a novel situation. However, we recognize that, according to the information criteria, the parameter estimates from the full fit scheme (option 1a) may be more appropriate for making predictions in novel data sets. As such, any readers interested in using the parameter estimates from the full fit scheme are invited to contact the lead author for the values.

*Single-axis scheme.* The single-axis scheme operated on the parameters controlling crown shape, and the statistical parameters$\sigma_{j},\varphi_{j},\rho_{j}$, only (i.e. all but the parameters $V_{bias,j}$). It reduces the interspecific variation in the crown shape model parameters to a single axis of variation, similarly to the way PCA analysis reduces multidimensional variation to a specified number of axes. Under this scheme, we assign each species a ‘trait score’ $T_{j}$, where $0<T_{j}<1$. The parameters for each species *j*, $\theta_{j}$ are then calculated from $T_{j}$ as follows:

$R_{0,j}$ = $[1-T_{j}].C_{0}(R_{0})$ + $T_{j}.C_{1}(R_{0})$

$R_{40,j}$ = $[1-T_{j}].C_{0}(R_{40})$ + $T_{j}.C_{1}(R_{40})$

$R_{us,j}$ = $[1-T_{j}].C_{0}(R_{us})$ + $T_{j}.C_{1}(R_{us})$

$M_{j}$ = $[1-T_{j}].C_{0}(M)$ + $T_{j}.C_{1}(M)$

$B_{j}$ = $[1-T_{j}].C_{0}(B)$ + $T_{j}.C_{1}(B)$

$V_{us,j}$ = $[1-T_{j}].C_{0}(V_{us})$ + $T_{j}.C_{1}(V_{us})$

$\rho_{j}$ = $[1-T_{j}].C_{0}(\rho)$ + $T_{j}.C_{1}(\rho)$

$\sigma_{j}$ = $[1-T_{j}].C_{0}(\sigma)$ + $T_{j}.C_{1}(\sigma)$

$\varphi_{j}$ = $[1-T_{j}].C_{0}(\varphi)$ + $T_{j}.C_{1}(\varphi)$ (S2.3)

where the parameters $C_{0}(R_{0})$ and $C_{1}(R_{0})$ are the values of $R_{0,j}$ when $T_{j}=0$ and $T_{j}=1$ respectively; and similarly for all other parameters $C_{0}(R_{40})$ … $C_{1}(\varphi)$. Any vector of parameter trait scores $T$($=T(1),T(2)....T(250)$ ) translates into a full parameter set $\theta$, as defined above. The parameters $C_{0}(R_{0})...C_{0}(\varphi)$ define the vector $C_{0}$ of parameters $\theta_{j}$ for a species with $T_{j}=0$; the parameters $C_{1}(R_{0})...C_{1}(\varphi)$ define a vector $C_{1}$ giving $\theta_{j}$ for $T_{j}=1$; and for any other species *j,* $\theta_{j}$ is an average of $C_{0}$ and $C_{1}$, weighted according to $T_{j}$.

Equation S3.3 is under-constrained, because there are multiple combinations of parameters $T_{j}$,$C_{0}(R_{0})...C_{0}(\varphi)$,$C_{1}(R_{0})...C_{1}(\varphi)$that yield identical parameter sets $\theta$, and hence likelihood function (e.g. swapping the vectors $C_{0}$ and $C_{1}$ and replacing each $T_{j}$ by $1-T_{j}$ yields an identical parameter set $\theta_{j}$ ). To resolve this problem, we fixed the parameters $C_{0}(R_{40})$ and $C_{1}(R_{40})$ at 0.5 and 10 m respectively, which encompass the reasonable range of variation for $R_{40}$. This ensured that each possible combination of $T_{j}$,$C_{0}(R_{0})...C_{0}(\varphi)$,$C_{1}(R_{0})...C_{1}(\varphi)$ corresponded to a unique $\theta$ and hence unique likelihood.

Once the species parameters are generated using eq. S3.3, the likelihood$L(Z|\theta)$, and hence the MLE estimates and confidence intervals, are estimated just as before (i.e. using eq. S3.1). Under this scheme, the parameters to be estimated are $C_{0}(R_{0})$ … $C_{1}(\varphi)$ (excluding$C_{0}(R_{40})$ or $C_{1}(R_{40})$ ), and the species trait scores $T$. The end result is a reduction in the number of free parameters from the 2250 (i.e. 2500 minus the species-specific $V_{bias,j}$) to 250 + 16 = 266. Moreover, under this scheme the vectors $C_{0}$ and $C_{1}$are expected to be determined mostly by the common species, because the large amount of data for these species allows only a limited flexibility in the eventual parameters $\theta_{j}$. Then, because the model parameters for each species *j* are an interpolation between $C_{0}$ and $C_{1}$, we expect this scheme to lead naturally to sensible combinations of crown shape parameters for rare species (which appeared to be the case: Fig. 2). Nevertheless, it is important to note that rare species will still tend to be assigned highly uncertain values of $T_{j}$, leading to corresponding uncertain in the eventual parameters $\theta_{j}$.

The single-axis scheme extends in an obvious way to the one-shape scheme: $T_{j}$ is fixed at 0 for every species; the parameters $C_{1}(R_{0})...C_{1}(\varphi)$then have no effect on the likelihood and so are fixed at arbitrary values, leaving $C_{0}(R_{0})...C_{0}(\varphi)$ as the only free parameters. Under this scheme, the vector $C_{0}(R_{0})...C_{0}(\varphi)$ defines a single crown shape and set of statistical parameters that applies to every species. Similarly, we tried setting $V_{bias,j}$ to single, free parameter that applied to all species; and we also tried fixing $V_{bias,j}$ at 0 (see above).

For all estimations, we found it necessary to fix the parameters $C_{0}(M)$ and $C_{1}(M)$ to the value 0.950. This was done because the single axis fit without the inclusion of $V_{bias,j}$(i.e. alternative 2c above) estimated values close to 0.95 for both $C_{0}(M)$ and $C_{1}(M)$, implying that it does not vary across species in way that is correlated with the other variables; and because there was evidence that the combined single-axis / $V_{bias,j}$ estimation failed to converge properly on the correct parameter distributions for species with large positive or negative$V_{bias,j}$, reflecting a (currently unexplored) complex interaction between the crown shape parameter $M_{j}$ and the depth bias $V_{bias,j}$, which evidently leads to a partially underconstrained problem.

**Appendix S3 references**

Burnham, K.P. & Anderson, D.R. (2004). Multimodel inference: understanding AIC and BIC in model selection. *Sociological Methods and Research*, **33**, 261 – 304.

Chib, S., & E. Greenberg. (1995). Understanding the Metropolis-Hasting algorithm. *The American Statistician*, **49**, 327–335.

Kuha, J. (2004). AIC and BIC: comparison of assumptions and performance.

Robert, C.P., and G. Casella. (1999). *Monte Carlo Statistical Methods*. Springer-Verlag, New York.
