# Supplementary material for: Crown Plasticity and Competition for Canopy Space: A New Spatially Implicit Model Parameterized for 250 North American Tree Species
Source: PLoS One. 2007 Sep 12;2(9):e870. doi: 10.1371/journal.pone.0000870 (PMC1964803; doi:10.1371/journal.pone.0000870)
Supplement: Table S1 — Summary of ITD parameter estimates the single axis scheme, with the additional free parameter Vbias,j. Parameters marked with * were fit as species-specific free parameters; for species j, parameters marked with {double dagger} depended only on the value of Tj. Parameter Mj was fixed at 0.95 for each species j, as shown. By definition, 5% of the species have values for parameter P above the 90% range for P, and 5% have values below the range. This interval was calculated for each parameter, either using only the 30 most common species, or all species, as shown. (0.06 MB DOC) [file pone.0000870.s004.doc]

| Parameter | Average MLE | 90% range  (30 most common) | 90% range  (all species) |
| --- | --- | --- | --- |
| ***** $T$ | 0.452 | 0.19, 0.58 | 0.22, 0.74 |
| **‡** $R_{0}$ | 1.834 | 1.06, 2.20 | 1.14, 2.67 |
| **‡** $R_{40}$ | 4.794 | 2.30, 5.98 | 2.56, 7.50 |
| **‡** $R_{us}$ | 2.391 | 1.42, 2.86 | 1.52, 3.45 |
| **‡** $B$ | 0.350 | 0.24, 0.40 | 0.25, 0.47 |
| **‡** $M$(fixed) | 0.95 | n/a | n/a |
| **‡** $V_{us}$ | 3.974 | 3.56, 4.17 | 3.60, 4.42 |
| ***** $V_{bias}$ | -0.410 | -3.79, 5.28 | -4.57, 4.63 |

**Table S4.2.** Parameters for converting the trait score for species *j*, $T_{j}$, to crown shape parameters (see eq. S2.3). Parameters marked fixed were not estimated, but fixed at the values given. Other parameters were fit as global free parameters, as part of the single-axis scheme. These values can be used with eq. S2.3 to assign species-specific crown shape parameters to species *j*, from the value of the trait score $T_{j}$ given in Table S4.3.

| Parameter *P* | $C_{0}(P)$ | $C_{1}(P)$ |
| --- | --- | --- |
| $R_{0}$ | 0.503 | 3.126 |
| $R_{40}$ | 0.5 (fixed) | 10.0 (fixed) |
| $R_{us}$ | 0.701 | 3.955 |
| $B$ | 0.196 | 0.511 |
| $M$ | 0.95 (fixed) | 0.95 (fixed) |
| $V_{us}$ | 2.551 | 4.106 |
