# Supplementary material for: Crown Plasticity and Competition for Canopy Space: A New Spatially Implicit Model Parameterized for 250 North American Tree Species
Source: PLoS One. 2007 Sep 12;2(9):e870. doi: 10.1371/journal.pone.0000870 (PMC1964803; doi:10.1371/journal.pone.0000870)
Supplement: Table S2 — Parameters for converting the trait score for species j, Tj, to crown shape parameters (see eq. S3.3). Parameters marked fixed were not estimated, but fixed at the values given. Other parameters were fit as global free parameters, as part of the single-axis scheme. These values can be used with eq. S3.3 to assign species-specific crown shape parameters to species j, from the value of the trait score Tj given in Table S3. (0.04 MB DOC) [file pone.0000870.s005.doc]

| Parameter *P* | $C_{0}(P)$ | $C_{1}(P)$ |
| --- | --- | --- |
| $R_{0}$ | 0.503 | 3.126 |
| $R_{40}$ | 0.5 (fixed) | 10.0 (fixed) |
| $R_{us}$ | 0.701 | 3.955 |
| $B$ | 0.196 | 0.511 |
| $M$ | 0.95 (fixed) | 0.95 (fixed) |
| $V_{us}$ | 2.551 | 4.106 |
