# Supplementary material for: Crown Plasticity and Competition for Canopy Space: A New Spatially Implicit Model Parameterized for 250 North American Tree Species
Source: PLoS One. 2007 Sep 12;2(9):e870. doi: 10.1371/journal.pone.0000870 (PMC1964803; doi:10.1371/journal.pone.0000870)
Supplement: Table S3 — ITD model parameters for the 250 tree species represented in the FHM data, together with height-dbh parameters for North American tree species not present in the FHM data. The height Hi (m) of a tree of species j, with dbhi in cm (or diameter at root collar, drci) can be calculated as Hi = 10ˆ[aj,dbh+bj.log10 (dbhi)], or as Hi = 10ˆ[aj,drc+bj.log10 (drci)] . The parameter Tj can be used in conjunction with Table S2 to generate species-specific crown shape parameters for species j. Vbias,j is a species-specific parameter required by the ITD model. The numeric code used to identify species in US Forest Service forest inventories is given. 95% confidence intervals are given for Tj and Vbias,j in parentheses. (0.46 MB DOC) [file pone.0000870.s006.doc]

|  |  |  |  | Height-dbh parameters | | | Crown shape parameters |
| --- | --- | --- | --- | --- | --- | --- | --- |
| Code | Common name | Scientific name | | $a_{j,dbh}$ | $a_{j,drc}$ | $b_{j}$ | $T_{j}$ $V_{bias,j}$ (m) |
| 10 | fir_sp. | *Abies* | *sp.* | 0.04542 | -0.1215 | 0.8117 | - |
| 11 | Pacific_silver_fir | *Abies* | *amabilis* | -0.09029 | -0.1208 | 0.8955 | 0.264 (0.247, 0.272) | -4.15 (-5.18, -2.52) |
| 12 | balsam_fir | *Abies* | *balsamea* | 0.1203 | -0.1211 | 0.783 | 0.278 (0.276, 0.286) | -4.01 (-4.19, -3.42) |
| 14 | Santa_Lucia_fir | *Abies* | *bracteata* | 0.04535 | -0.1197 | 0.8127 | - |
| 15 | white_fir | *Abies* | *concolor* | 0.03303 | -0.1204 | 0.7735 | 0.236 (0.236, 0.244) | 2.326 (2.134, 2.980) |
| 16 | Fraser_fir | *Abies* | *fraseri* | 0.0954 | -0.1206 | 0.8304 | - |
| 17 | grand_fir | *Abies* | *grandis* | 0.04658 | -0.1208 | 0.7875 | 0.292 (0.291, 0.308) | -4.74 (-5.30, -1.91) |
| 18 | corkbark_fir | *Abies* | *lasiocarpa*  *var._arizonica* | 0.007404 | -0.1211 | 0.8805 | 0.177 (0.166, 0.216) | -4.35 (-5.33, 2.743) |
| 19 | subalpine_fir | *Abies* | *lasiocarpa* | 0.01819 | -0.1206 | 0.8281 | 0.181 (0.180, 0.189) | -1.26 (-1.93, -1.08) |
| 20 | California_red_fir | *Abies* | *magnifica* | 0.0511 | -0.1193 | 0.7997 | 0.181 (0.168, 0.182) | 2.754 (2.668, 3.933) |
| 21 | Shasta_red_fir | *Abies* | *magnifica*  *var.shastensis* | 0.00518 | -0.1211 | 0.805 | 0.193 (0.157, 0.201) | 2.440 (-1.26, 3.214) |
| 22 | noble_fir | *Abies* | *procera* | -0.04889 | -0.1209 | 0.8581 | 0.286 (0.261, 0.309) | -0.75 (-1.85, 1.382) |
| 41 | Port-Orford-cedar | *Chamaecyparis* | *lawsoniana* | 0.2136 | -0.1208 | 0.6974 | 0.196 (0.187, 0.212) | -2.54 (-3.19, -2.34) |
| 42 | Alaska_yellow-cedar | *Chamaecyparis* | *nootkatensis* | 0.1417 | -0.1203 | 0.6981 | 0.202 (0.172, 0.276) | -3.69 (-7.90, -0.35) |
| 43 | Atlantic_white-cedar | *Chamaecyparis* | *thyoides* | 0.2216 | -0.1193 | 0.7052 | 0.959 (0.632, 0.992) | 0.450 (-1.11, 0.456) |
| 50 | cypress | *Cupressus* | *sp.* | 0.1507 | -0.1198 | 0.633 | - |
| 51 | Arizona_cypress | *Cupressus* | *arizonica* | 0.1278 | -0.1212 | 0.5825 | 0.766 (0.027, 0.983) | -3.59 (-7.71, 7.636) |
| 52 | Baker_cypress | *Cupressus* | *bakeri* | 0.1512 | -0.1216 | 0.6328 | - |
| 53 | Tecate_cypress | *Cupressus* | *guadalupensis*  *var._forbesii* | 0.1511 | -0.121 | 0.6332 | - |
| 54 | Monterey_cypress | *Cupressus* | *macrocarpa* | 0.1508 | -0.1211 | 0.6332 | 0.813 (0.045, 0.987) | -4.08 (-7.44, 4.808) |
| 55 | Sargent_cypress | *Cupressus* | *sargentii* | 0.1506 | -0.1208 | 0.6338 | - |
| 57 | Juniperus_sp. | *Juniperus* | *sp.* | 0.2223 | -0.1444 | 0.5253 | 0.288 (0.227, 0.603) | -6.33 (-7.73, 0.833) |
| 58 | Pinchot_juniper | *Juniperus* | *pinchotii* | 0.2369 | -0.1443 | 0.5471 | - |
| 59 | redberry_juniper | *Juniperus* | *coahuilensis* | 0.2375 | -0.1216 | 0.5534 | 0.740 (0.033, 0.992) | -5.41 (-7.32, 7.311) |
| 60 | common_juniper | *Juniperus* | *communis* | 0.2372 | -0.1437 | 0.5475 | - |
| 61 | Ashe_juniper | *Juniperus* | *ashei* | 0.2366 | -0.1436 | 0.5478 | - |
| 62 | California_juniper | *Juniperus* | *californica* | 0.2372 | -0.2514 | 0.5088 | - |
| 63 | alligator_juniper | *Juniperus* | *deppeana* | 0.2376 | -0.1031 | 0.594 | 0.167 (0.064, 0.949) | 3.311 (-7.98, 6.794) |
| 64 | western_juniper | *Juniperus* | *occidentalis* | 0.2167 | -0.1443 | 0.5037 | 0.015 (0.000, 0.158) | 2.884 (2.453, 3.271) |
| 65 | Utah_juniper | *Juniperus* | *osteosperma* | 0.2369 | -0.1376 | 0.4802 | - |
| 66 | Rocky_Mountain  juniper | *Juniperus* | *scopulorum* | 0.2368 | -0.1522 | 0.5566 | 0.716 (0.028, 0.967) | 6.081 (-7.81, 7.690) |
| 67 | southern_redcedar | *Juniperus* | *silicicola* | 0.2869 | -0.1455 | 0.5825 | 0.305 (0.211, 0.388) | -6.49 (-6.96, 1.187) |
| 68 | eastern_redcedar | *Juniperus* | *virginiana* | 0.3195 | -0.1442 | 0.5537 | 0.380 (0.364, 0.391) | -3.32 (-3.99, -2.85) |
| 69 | oneseed_juniper | *Juniperus* | *monosperma* | 0.2719 | -0.1228 | 0.482 | 0.394 (0.090, 0.977) | -5.91 (-7.79, 7.753) |
| 70 | larch_(introduced) | *Larix* | *sp.* | 0.2406 | -0.12 | 0.69 | 0.389 (0.350, 0.643) | -2.96 (-7.74, 6.538) |
| 71 | tamarack_(native) | *Larix* | *laricina* | 0.3045 | -0.1195 | 0.6559 | 0.308 (0.308, 0.333) | -0.44 (-1.02, -0.18) |
| 72 | subalpine_larch | *Larix* | *lyallii* | 0.1993 | -0.1201 | 0.6666 | 0.225 (0.182, 0.266) | 4.170 (-2.25, 5.742) |
| 73 | western_larch | *Larix* | *occidentalis* | 0.316 | -0.1208 | 0.7169 | 0.344 (0.316, 0.344) | 7.975 (7.687, 7.992) |
| 81 | incense-cedar | *Calocedrus* | *decurrens* | 0.05112 | -0.1211 | 0.7242 | 0.240 (0.236, 0.252) | 0.050 (-0.14, 0.926) |
| 90 | spruce_sp. | *Picea* | *sp.* | 0.1509 | -0.1213 | 0.7353 | 0.536 (0.081, 0.963) | 2.876 (-7.30, 6.346) |
| 91 | Norway_spruce | *Picea* | *abies* | 0.2259 | -0.1213 | 0.7156 | 0.282 (0.268, 0.318) | -4.34 (-4.46, -3.32) |
| 92 | Brewer_spruce | *Picea* | *breweriana* | 0.1505 | -0.1219 | 0.7355 | - |
| 93 | Engelmann  spruce | *Picea* | *engelmannii* | 0.06969 | -0.1234 | 0.7796 | 0.208 (0.203, 0.211) | 2.971 (2.345, 2.972) |
| 94 | white_spruce | *Picea* | *glauca* | 0.1404 | -0.1206 | 0.7354 | 0.278 (0.272, 0.296) | -3.85 (-4.00, -3.06) |
| 95 | black-spruce | *Picea* | *mariana* | 0.175 | -0.1214 | 0.7487 | 0.201 (0.200, 0.212) | -0.86 (-1.23, -0.45) |
| 96 | blue_spruce | *Picea* | *pungens* | 0.1241 | -0.1208 | 0.7326 | 0.227 (0.197, 0.255) | -3.39 (-4.27, -0.93) |
| 97 | red-spruce | *Picea* | *rubens* | 0.1309 | -0.1225 | 0.7555 | 0.324 (0.319, 0.338) | -2.42 (-2.75, -2.08) |
| 98 | Sitka_spruce | *Picea* | *sitchensis* | 0.1663 | -0.1203 | 0.724 | 0.316 (0.296, 0.328) | -5.51 (-6.87, -3.51) |
| 101 | whitebark_pine | *Pinus* | *albicaulis* | 0.09986 | -0.1386 | 0.708 | 0.290 (0.243, 0.305) | 3.726 (3.045, 4.703) |
| 102 | bristlecone_pine | *Pinus* | *aristata* | 0.1664 | -0.1405 | 0.5661 | 0.320 (0.272, 0.329) | -3.02 (-7.85, -1.36) |
| 103 | knobcone_pine | *Pinus* | *attenuata* | 0.1899 | -0.1392 | 0.6954 | 0.329 (0.263, 0.339) | 2.308 (1.481, 2.720) |
| 104 | foxtail_pine | *Pinus* | *balfouriana* | 0.1771 | -0.1394 | 0.6896 | - |
| 105 | jack_pine | *Pinus* | *banksiana* | 0.223 | -0.1403 | 0.6828 | 0.342 (0.342, 0.381) | 1.559 (0.967, 2.348) |
| 106 | common_pinyon | *Pinus* | *edulis* | 0.1773 | -0.188 | 0.6372 | - |
| 107 | sand-pine | *Pinus* | *clausa* | 0.1756 | -0.1402 | 0.6842 | - |
| 108 | lodgepole_pine | *Pinus* | *contorta_var*  *latifolia* | 0.1645 | -0.1397 | 0.77 | 0.235 (0.232, 0.238) | 5.290 (5.177, 5.546) |
| 109 | Coulter_pine | *Pinus* | *coulteri* | 0.1782 | -0.1398 | 0.6907 | 0.248 (0.226, 0.433) | 7.961 (4.578, 7.968) |
| 110 | shortleaf_pine | *Pinus* | *echinata* | 0.2356 | -0.1404 | 0.7331 | 0.407 (0.394, 0.420) | 2.768 (2.347, 3.094) |
| 111 | slash-pine | *Pinus* | *elliottii* | 0.1785 | -0.1391 | 0.7925 | 0.406 (0.394, 0.413) | 4.598 (4.186, 4.848) |
| 112 | Apache_pine | *Pinus* | *engelmannii* | 0.1364 | -0.1398 | 0.6634 | - |
| 113 | limber_pine | *Pinus* | *flexilis* | 0.1703 | -0.1392 | 0.5895 | 0.314 (0.266, 0.334) | 1.871 (-6.82, 1.947) |
| 114 | southwestern_  white_pine_ | *Pinus* | *strobiformus* | 0.1865 | -0.1392 | 0.6828 | 0.038 (0.028, 0.987) | -6.53 (-7.81, 7.473) |
| 115 | spruce_pine | *Pinus* | *glabra* | 0.2673 | -0.14 | 0.7058 | 0.426 (0.395, 0.546) | 3.137 (0.291, 4.067) |
| 116 | Jeffrey_pine | *Pinus* | *jeffreyi* | 0.1773 | -0.1402 | 0.6977 | 0.292 (0.271, 0.291) | 3.371 (2.713, 3.975) |
| 117 | sugar_pine | *Pinus* | *lambertiana* | 0.1509 | -0.1401 | 0.7349 | 0.365 (0.356, 0.376) | 3.803 (3.630, 4.391) |
| 118 | Chihuahua_pine | *Pinus* | *leiophylla_var*  *chihuahuana* | 0.09448 | -0.1396 | 0.6079 | - |
| 119 | western_white-pine | *Pinus* | *monticola* | 0.04624 | -0.1404 | 0.8043 | 0.172 (0.033, 0.969) | 0.622 (-7.58, 6.950) |
| 120 | bishop_pine | *Pinus* | *muricata* | 0.1776 | -0.1396 | 0.6901 | - |
| 121 | longleaf_pine | *Pinus* | *palustris* | 0.2001 | -0.1404 | 0.7659 | 0.462 (0.443, 0.492) | 4.883 (4.476, 5.224) |
| 122 | ponderosa_pine | *Pinus* | *ponderosa* | 0.06788 | -0.1403 | 0.7441 | 0.314 (0.306, 0.318) | 4.614 (4.335, 4.756) |
| 123 | Table_Mountain-pine | *Pinus* | *pungens* | 0.2218 | -0.1394 | 0.6883 | 0.441 (0.435, 0.519) | -2.38 (-3.25, -1.38) |
| 124 | Monterey_pine | *Pinus* | *radiata* | 0.1776 | -0.1384 | 0.6904 | - |
| 125 | red_pine | *Pinus* | *resinosa* | 0.2567 | -0.1408 | 0.6522 | 0.287 (0.283, 0.290) | -1.52 (-1.73, -1.17) |
| 126 | pitch_pine | *Pinus* | *rigida* | 0.197 | -0.1412 | 0.6784 | 0.485 (0.428, 0.498) | 0.245 (-0.24, 0.908) |
| 127 | gray_pine | *Pinus* | *sabiniana* | 0.1771 | -0.1407 | 0.6899 | 0.409 (0.378, 0.421) | 4.624 (4.183, 5.933) |
| 128 | pond_pine | *Pinus* | *serotina* | 0.2432 | -0.1395 | 0.7453 | 0.483 (0.422, 0.504) | 4.897 (4.718, 5.355) |
| 129 | eastern_white_pine | *Pinus* | *strobus* | 0.228 | -0.1407 | 0.6755 | 0.417 (0.415, 0.419) | -1.80 (-1.89, -1.49) |
| 130 | Scotch_pine | *Pinus* | *sylvestris* | 0.2173 | -0.1394 | 0.6544 | 0.367 (0.333, 0.385) | -1.11 (-2.00, -0.38) |
| 131 | loblolly_pine | *Pinus* | *taeda* | 0.2401 | -0.1397 | 0.7302 | 0.355 (0.353, 0.359) | 1.711 (1.609, 1.790) |
| 132 | Virginia_pine | *Pinus* | *virginiana* | 0.2829 | -0.14 | 0.691 | 0.427 (0.416, 0.437) | -0.29 (-0.45, -0.04) |
| 133 | singleleaf_pinyon | *Pinus* | *monophylla* | 0.1774 | -0.292 | 0.7446 | 0.538 (0.037, 0.933) | -4.51 (-7.11, 7.383) |
| 134 | border_pinyon | *Pinus* | *discolor* | 0.1777 | -0.07711 | 0.6228 | - |
| 135 | Arizona_pine | *Pinus* | *ponderosa_var*  *arizonica* | 0.1214 | -0.1417 | 0.6273 | - |
| 136 | Austrian_pine | *Pinus* | *nigra* | 0.205 | -0.14 | 0.7224 | - |
| 137 | Washoe_pine | *Pinus* | *washoensis* | 0.1783 | -0.1387 | 0.6898 | - |
| 138 | four-needle_pinyon | *Pinus* | *quadrifolia* | 0.1777 | -0.1398 | 0.6899 | - |
| 139 | Torrey_pine | *Pinus* | *torreyana* | 0.1774 | -0.1399 | 0.6902 | - |
| 140 | Mexican_pinyon_pine | *Pinus* | *cembroides* | 0.1771 | -0.03547 | 0.583 | - |
| 142 | Great_Basin  bristlecone_pine | *Pinus* | *longaeva* | 0.1398 | -0.141 | 0.5744 | - |
| 143 | Arizona_pinyon_pine | *Pinus* | *monophylla_var*  *fallax* | 0.1769 | -0.1721 | 0.7293 | - |
| 201 | bigcone_Douglas-fir | *Pseudotsuga* | *macrocarpa* | 0.1528 | -0.1232 | 0.7315 | - |
| 202 | Douglas-fir | *Pseudotsuga* | *menziesii*  *menziesii* | 0.1426 | -0.1224 | 0.7589 | 0.335 (0.333, 0.337) | 2.448 (2.328, 2.459) |
| 211 | redwood | *Sequoia* | *sempervirens* | 0.1641 | -0.1212 | 0.6887 | 0.355 (0.328, 0.368) | -0.78 (-1.87, 0.304) |
| 212 | giant_sequoia | *Sequoiadendron* | *giganteum* | 0.1646 | -0.121 | 0.6882 | - |
| 221 | baldcypress | *Taxodium* | *distichum* | 0.1858 | -0.1191 | 0.7323 | 0.361 (0.350, 0.378) | -2.38 (-3.07, -2.31) |
| 222 | pondcypress | *Taxodium* | *distichum_var*  *nutans* | 0.198 | -0.1194 | 0.7415 | 0.623 (0.587, 0.723) | 4.663 (2.373, 5.847) |
| 231 | Pacific_yew | *Taxus* | *brevifolia* | 0.1519 | -0.1208 | 0.5919 | 0.803 (0.474, 0.904) | 4.703 (-2.04, 4.597) |
| 241 | northern_white-cedar | *Thuja* | *occidentalis* | 0.2302 | -0.1199 | 0.6194 | 0.251 (0.245, 0.259) | -1.84 (-2.07, -1.52) |
| 242 | western_redcedar | *Thuja* | *plicata* | 0.2029 | -0.1196 | 0.6842 | 0.334 (0.331, 0.349) | -6.29 (-7.71, -5.29) |
| 251 | California_torrey  (nutmeg) | *Torreya* | *californica* | 0.1646 | -0.1209 | 0.6895 | 0.445 (0.072, 0.894) | 4.621 (-7.03, 7.312) |
| 252 | Florida_torreya | *Torreya* | *taxifolia* | 0.164 | -0.1216 | 0.6888 | - |
| 260 | hemlock_sp. | *Tsuga* | *sp.* | 0.1454 | -0.1218 | 0.7112 | 0.426 (0.389, 0.522) | -6.75 (-7.95, -3.90) |
| 261 | eastern_hemlock | *Tsuga* | *canadensis* | 0.2893 | -0.1222 | 0.6058 | 0.464 (0.459, 0.477) | -4.80 (-5.04, -4.31) |
| 262 | Carolina_hemlock | *Tsuga* | *caroliniana* | 0.1948 | -0.1198 | 0.7325 | - |
| 263 | western_hemlock | *Tsuga* | *heterophylla* | 0.1829 | -0.1208 | 0.7327 | 0.337 (0.329, 0.340) | -2.71 (-2.94, -2.31) |
| 264 | mountain_hemlock | *Tsuga* | *mertensiana* | -0.05736 | -0.1211 | 0.8255 | 0.209 (0.195, 0.212) | 2.580 (1.566, 2.818) |
| 270 | Australian_pine | *Casuarina* | *sp.* | 0.1636 | -0.1227 | 0.6881 | - |
| 299 | uknown_dead_conifer | *dead_conifer* | *sp.* | 0.1639 | -0.1215 | 0.6887 | - |
| 300 | acacia_sp. | *Acacia* | *sp.* | 0.4063 | -0.05921 | 0.5516 | - |
| 310 | maple_sp. | *Acer* | *sp.* | 0.4863 | 0.1891 | 0.5679 | 0.406 (0.063, 0.966) | -1.52 (-6.81, 7.612) |
| 311 | Florida_maple | *Acer* | *barbatum* | 0.4258 | 0.1903 | 0.5844 | 0.639 (0.513, 0.971) | 0.479 (-4.54, 0.492) |
| 312 | bigleaf_maple | *Acer* | *macrophyllum* | 0.4854 | 0.1901 | 0.5231 | 0.493 (0.473, 0.506) | -1.81 (-3.85, -1.06) |
| 313 | boxelder | *Acer* | *negundo* | 0.5276 | 0.1906 | 0.4269 | 0.646 (0.556, 0.646) | -0.54 (-1.51, -0.52) |
| 314 | black_maple | *Acer* | *nigrum* | 0.5096 | 0.1897 | 0.5369 | 0.966 (0.528, 0.979) | -1.19 (-6.78, 1.370) |
| 315 | striped_maple | *Acer* | *pensylvanicum* | 0.4352 | 0.1893 | 0.5372 | 0.674 (0.560, 0.856) | -1.36 (-2.29, -0.38) |
| 316 | red_maple | *Acer* | *rubrum* | 0.4769 | 0.1905 | 0.5641 | 0.536 (0.532, 0.536) | -1.35 (-1.53, -1.28) |
| 317 | silver_maple | *Acer* | *saccharinum* | 0.4801 | 0.1901 | 0.5341 | 0.486 (0.483, 0.517) | -0.06 (-0.60, 0.196) |
| 318 | sugar_maple | *Acer* | *saccharum* | 0.4742 | 0.1891 | 0.5743 | 0.560 (0.557, 0.562) | -2.44 (-2.62, -2.31) |
| 319 | mountain_maple | *Acer* | *spicatum* | 0.351 | 0.1894 | 0.5975 | 0.841 (0.602, 0.998) | -0.77 (-7.64, -0.21) |
| 320 | Norway_maple | *Acer* | *platanoides* | 0.456 | 0.1898 | 0.5077 | 0.600 (0.050, 0.975) | -5.48 (-7.52, 7.071) |
| 321 | Rocky_Mountain  maple | *Acer* | *glabrum* | 0.4543 | 0.4822 | 0.3276 | 0.778 (0.013, 0.945) | -1.17 (-7.53, 6.983) |
| 322 | bigtooth_maple | *Acer* | *grandidentatum* | 0.4535 | 0.06672 | 0.6452 | 0.135 (0.037, 0.946) | -0.51 (-7.56, 7.118) |
| 323 | chalk_maple | *Acer* | *leucoderme* | 0.4262 | 0.1902 | 0.5812 | - |
| 330 | buckeye_or  horsechestnut | *Aesculus* | *sp.* | 0.3476 | -0.05876 | 0.6341 | - |
| 331 | Ohio_buckeye | *Aesculus* | *glabra* | 0.2854 | -0.05975 | 0.6303 | 0.446 (0.390, 0.559) | -4.89 (-7.78, -0.18) |
| 332 | yellow_buckeye | *Aesculus* | *octandra* | 0.3314 | -0.05865 | 0.655 | 0.907 (0.698, 0.979) | 0.695 (-1.41, 0.788) |
| 333 | California_buckeye | *Aesculus* | *californica* | 0.3492 | -0.0577 | 0.6169 | 0.372 (0.322, 0.490) | -6.58 (-7.74, 0.050) |
| 334 | Texas_buckeye | *Aesculus* | *glabra_var*  *arguta* | 0.3298 | -0.05798 | 0.6094 | - |
| 341 | ailanthus | *Ailanthus* | *altissima* | 0.4158 | -0.06065 | 0.6115 | 0.588 (0.510, 0.908) | -0.40 (-2.89, 1.613) |
| 345 | mimosa_or_silktree | *Albizia* | *julibrissin* | 0.4686 | -0.06105 | 0.536 | - |
| 351 | red_alder | *Alnus* | *rubra* | 0.392 | -0.05873 | 0.6299 | 0.437 (0.417, 0.465) | 3.257 (3.173, 3.980) |
| 352 | white_alder | *Alnus* | *rhombifolia* | 0.294 | -0.05969 | 0.5838 | 0.446 (0.412, 0.520) | 3.602 (2.806, 5.438) |
| 355 | European_alder | *Alnus* | *glutinosa* | 0.3913 | -0.05817 | 0.5995 | 0.566 (0.396, 0.873) | -6.22 (-7.46, 3.299) |
| 356 | serviceberry | *Amelanchier* | *sp.* | 0.3863 | -0.05938 | 0.5775 | 0.534 (0.421, 0.577) | -2.15 (-5.08, -2.15) |
| 361 | Pacific_madrone | *Arbutus* | *menziesii* | 0.2944 | -0.05729 | 0.6054 | 0.357 (0.345, 0.381) | 2.694 (2.314, 3.038) |
| 367 | pawpaw | *Asimina* | *triloba* | 0.3911 | -0.05744 | 0.5464 | 0.381 (0.343, 0.821) | -7.50 (-7.87, 1.377) |
| 370 | birch_sp. | *Betula* | *sp.* | 0.4521 | -0.05974 | 0.5272 | 0.499 (0.416, 0.547) | -3.12 (-7.81, -1.12) |
| 371 | yellow_birch | *Betula* | *alleghaniensis* | 0.5297 | -0.05898 | 0.471 | 0.592 (0.566, 0.592) | -2.62 (-3.04, -2.37) |
| 372 | sweet_birch | *Betula* | *lenta* | 0.492 | -0.05886 | 0.5399 | 0.571 (0.540, 0.582) | -2.79 (-2.79, -2.20) |
| 373 | river_birch | *Betula* | *nigra* | 0.5059 | -0.05804 | 0.5297 | 0.605 (0.525, 0.648) | -0.74 (-1.99, 0.347) |
| 374 | water_birch | *Betula* | *occidentalis* | 0.4538 | -0.05893 | 0.5187 | - |
| 375 | paper_birch | *Betula* | *papyrifera* | 0.4423 | -0.05837 | 0.5634 | 0.435 (0.431, 0.448) | -1.11 (-1.20, -0.72) |
| 376 | western  paper_birch | *Betula* | *papyrifera_var*  *commutata* | 0.461 | -0.05815 | 0.5283 | 0.275 (0.231, 0.553) | -6.49 (-7.69, 1.535) |
| 378 | northwestern  paper_birch | *Betula* | *papyrifera_var*  *subcordata* | 0.4612 | -0.05911 | 0.529 | - |
| 379 | gray_birch | *Betula* | *populifolla* | 0.4551 | -0.05965 | 0.5319 | 0.371 (0.344, 0.461) | -2.53 (-3.14, -1.38) |
| 381 | chittamwood_  or_gum_bumelia | *Bumelia* | *lanuginosa* | 0.4455 | -0.06075 | 0.465 | 0.499 (0.416, 0.547) | -3.12 (-7.81, -1.12) |
| 391 | American_hornbeam  or_musclewood | *Carpinus* | *caroliniana* | 0.5218 | -0.05893 | 0.4359 | 0.719 (0.661, 0.854) | -2.83 (-3.81, -1.96) |
| 400 | hickory_sp. | *Carya* | *sp.* | 0.3745 | -0.05935 | 0.6142 | 0.561 (0.510, 0.561) | -1.31 (-2.26, -1.06) |
| 401 | water_hickory | *Carya* | *aquatica* | 0.4199 | -0.05915 | 0.5596 | 0.955 (0.731, 0.997) | 3.351 (0.543, 6.162) |
| 402 | bitternut_hickory | *Carya* | *cordiformis* | 0.3579 | -0.05922 | 0.6582 | 0.577 (0.531, 0.604) | -0.52 (-1.45, -0.16) |
| 403 | pignut_hickory | *Carya* | *glabra* | 0.3502 | -0.0583 | 0.6749 | 0.564 (0.557, 0.592) | -0.95 (-1.20, -0.40) |
| 404 | pecan | *Carya* | *illinoensis* | 0.4088 | -0.05929 | 0.5985 | 0.715 (0.603, 0.953) | 0.011 (-1.99, 5.354) |
| 405 | shellbark_hickory | *Carya* | *laciniosa* | 0.3228 | -0.05945 | 0.6823 | 0.486 (0.428, 0.525) | -5.73 (-7.08, -2.70) |
| 406 | nutmeg_hickory | *Carya* | *myristiciformis* | 0.3792 | -0.06027 | 0.6212 | - |
| 407 | shagbark_hickory | *Carya* | *ovata* | 0.3742 | -0.06024 | 0.6423 | 0.486 (0.468, 0.507) | -1.37 (-2.27, -1.24) |
| 408 | black_hickory | *Carya* | *texana* | 0.3222 | -0.06009 | 0.6448 | 0.686 (0.590, 0.763) | -0.90 (-1.52, 0.276) |
| 409 | mockernut_hickory | *Carya* | *tomentosa* | 0.3633 | -0.05892 | 0.6506 | 0.599 (0.593, 0.615) | -0.98 (-1.50, -0.65) |
| 410 | sand_hickory | *Carya* | *pallida* | 0.401 | -0.05983 | 0.6379 | 0.128 (0.016, 0.832) | -1.50 (-6.56, 6.186) |
| 421 | American_chestnut | *Castanea* | *dentata* | 0.4201 | -0.05873 | 0.5494 | 0.457 (0.331, 0.806) | -2.62 (-5.98, 7.045) |
| 422 | Allegheny_chinkapin | *Castanea* | *pumila* | 0.455 | -0.06017 | 0.572 | - |
| 423 | Ozark_chinkapin | *Castanea* | *ozarkensis* | 0.4227 | -0.05954 | 0.5584 | - |
| 430 | western  chinkapin_sp. | *Chrysolepis* | *sp.* | 0.3396 | -0.06123 | 0.6171 | - |
| 431 | giant_or  golden_chinkapin | *Chrysolepis* | *chrysophylla* | 0.2263 | -0.05854 | 0.678 | 0.551 (0.418, 0.825) | 1.979 (1.036, 5.090) |
| 450 | catalpa_sp. | *Catalpa* | *sp.* | 0.4505 | -0.05947 | 0.5197 | 0.721 (0.022, 0.964) | -6.18 (-7.50, 7.578) |
| 451 | southern_catalpa | *Catalpa* | *bignonioides* | 0.4647 | -0.05865 | 0.4857 | 0.461 (0.021, 0.973) | -5.04 (-7.16, 7.541) |
| 452 | northern_catalpa | *Catalpa* | *speciosa* | 0.4405 | -0.0596 | 0.4915 | 0.373 (0.228, 0.772) | -3.95 (-7.42, 7.394) |
| 460 | hackberry_sp. | *Celtis* | *sp.* | 0.4213 | -0.05942 | 0.5994 | 0.507 (0.467, 0.642) | -4.60 (-6.35, -0.74) |
| 461 | sugarberry | *Celtis* | *laevigata* | 0.423 | -0.05818 | 0.5687 | 0.670 (0.576, 0.786) | -0.31 (-2.50, 0.794) |
| 462 | hackberry | *Celtis* | *occidentalis* | 0.3411 | -0.05958 | 0.6015 | 0.599 (0.586, 0.618) | -1.91 (-2.38, -0.86) |
| 463 | netleaf_hackberry | *Celtis* | *reticulata* | 0.3995 | -0.06015 | 0.5802 | - |
| 471 | eastern_redbud | *Cercis* | *canadensis* | 0.4583 | -0.06018 | 0.4644 | 0.698 (0.449, 0.847) | -1.18 (-4.80, -0.07) |
| 475 | curlleaf_mountain  mahogany | *Cercocarpus* | *ledifolius* | 0.4058 | -0.08208 | 0.5224 | 0.222 (0.042, 0.984) | -2.46 (-7.26, 7.557) |
| 476 | true_mountain  mahogany | *Cercocarpus* | *montanus* | 0.406 | -0.07093 | 0.5369 | - |
| 477 | hairy_mountain  mahogany | *Cercocarpus* | *montanus_var*  *paucidentatus* | 0.4065 | -0.07072 | 0.5369 | - |
| 478 | birchleaf_mountain  mahogany | *Cercocarpus* | *montanus_var*  *glaber* | 0.406 | -0.07033 | 0.5366 | - |
| 479 | littleleaf_mountain  mahogany | *Cercocarpus* | *intricatus* | 0.4063 | -0.07038 | 0.5369 | - |
| 481 | yellowwood | *Cladrastis* | *kentukea* | 0.419 | -0.05919 | 0.5754 | 0.445 (0.018, 0.983) | 3.332 (-7.41, 6.728) |
| 491 | flowering_dogwood | *Cornus* | *florida* | 0.4421 | -0.05873 | 0.4583 | 0.624 (0.568, 0.713) | -3.22 (-5.04, -2.69) |
| 492 | Pacific_dogwood | *Cornus* | *nuttallii* | 0.4243 | -0.05798 | 0.5305 | 0.981 (0.196, 0.981) | 7.113 (-7.09, 7.611) |
| 500 | hawthorn_sp. | *Crataegus* | *sp.* | 0.4804 | -0.05938 | 0.3798 | 0.526 (0.436, 0.612) | -5.33 (-7.41, -0.25) |
| 501 | cockspur_hawthorn | *Crataegus* | *crus-galli* | 0.4104 | -0.05887 | 0.4403 | 0.593 (0.037, 0.930) | -0.03 (-7.78, 6.811) |
| 502 | downy_hawthorn | *Crataegus* | *mollis* | 0.3677 | -0.05935 | 0.5715 | 0.016 (0.089, 0.966) | -2.55 (-7.98, 7.639) |
| 510 | eucalyptus | *Eucalyptus* | *sp.* | 0.4058 | -0.05952 | 0.5523 | - |
| 521 | common_persimmon | *Diospyros* | *virginiana* | 0.3575 | -0.05915 | 0.6226 | 0.526 (0.453, 0.577) | -0.41 (-0.65, 0.313) |
| 531 | American_beech | *Fagus* | *grandifolia* | 0.3989 | -0.05996 | 0.5954 | 0.626 (0.622, 0.638) | -3.91 (-3.94, -3.46) |
| 540 | ash_sp. | *Fraxinus* | *sp.* | 0.4387 | -0.05852 | 0.5568 | 0.531 (0.490, 0.596) | -2.76 (-3.36, -0.99) |
| 541 | white_ash | *Fraxinus* | *americana* | 0.4466 | -0.0581 | 0.5645 | 0.551 (0.542, 0.557) | -0.31 (-0.58, -0.13) |
| 542 | Oregon_ash | *Fraxinus* | *latifolia* | 0.4827 | -0.05902 | 0.5152 | 0.466 (0.444, 0.656) | -0.03 (-1.66, 3.717) |
| 543 | black_ash | *Fraxinus* | *nigra* | 0.4782 | -0.05922 | 0.524 | 0.374 (0.362, 0.393) | -0.16 (-0.61, 0.133) |
| 544 | green_ash | *Fraxinus* | *pennsylvanica* | 0.4525 | -0.05826 | 0.534 | 0.511 (0.488, 0.544) | -0.66 (-1.07, -0.31) |
| 545 | pumpkin_ash | *Fraxinus* | *profunda* | 0.4486 | -0.0592 | 0.5362 | 0.073 (0.048, 0.962) | -2.04 (-7.55, 7.876) |
| 546 | blue_ash | *Fraxinus* | *quadrangulata* | 0.4345 | -0.05896 | 0.5701 | 0.532 (0.398, 0.903) | 2.933 (-0.75, 4.911) |
| 547 | velvet_ash | *Fraxinus* | *velutina* | 0.3443 | -0.05945 | 0.4884 | - |
| 548 | Carolina_ash | *Fraxinus* | *caroliniana* | 0.4321 | -0.05735 | 0.5466 | - |
| 551 | waterlocust | *Gleditsia* | *aquatica* | 0.4241 | -0.05874 | 0.5368 | 0.669 (0.420, 0.991) | -5.70 (-6.23, 6.250) |
| 552 | honeylocust | *Gleditsia* | *triacanthos* | 0.4269 | -0.05817 | 0.5303 | 0.573 (0.504, 0.644) | -2.60 (-4.10, -1.03) |
| 555 | loblolly-bay | *Gordonia* | *lasianthus* | 0.3452 | -0.05763 | 0.4855 | 0.577 (0.207, 0.656) | 3.918 (0.902, 7.243) |
| 571 | Kentucky_coffeetree | *Gymnocladus* | *dioicus* | 0.4654 | -0.05953 | 0.5639 | 0.861 (0.475, 0.886) | -2.82 (-4.50, 3.352) |
| 580 | silverbell_sp. | *Halesia* | *sp.* | 0.4096 | -0.06133 | 0.6152 | 0.612 (0.367, 0.690) | -3.64 (-6.93, 0.221) |
| 591 | American_holly | *Ilex* | *opaca* | 0.2903 | -0.06057 | 0.6099 | 0.462 (0.413, 0.475) | -7.13 (-7.92, -6.15) |
| 600 | walnut_sp. | *Juglans* | *sp.* | 0.3463 | -0.0588 | 0.4552 | 0.435 (0.297, 0.696) | -0.40 (-6.77, 5.852) |
| 601 | butternut | *Juglans* | *cinerea* | 0.4423 | -0.05786 | 0.5323 | 0.506 (0.411, 0.625) | -2.65 (-3.61, -0.42) |
| 602 | black_walnut | *Juglans* | *nigra* | 0.3885 | -0.05877 | 0.5843 | 0.653 (0.602, 0.687) | 0.511 (0.187, 0.879) |
| 603 | California  black_walnut | *Juglans* | *hindsii* | 0.3966 | -0.05815 | 0.5304 | - |
| 604 | southern_California  black_walnut | *Juglans* | *californica* | 0.3978 | -0.05959 | 0.5306 | - |
| 605 | Texas_walnut | *Juglans* | *microcarpa* | 0.3973 | -0.05872 | 0.5307 | - |
| 611 | sweetgum | *Liquidambar* | *styraciflua* | 0.3699 | -0.06079 | 0.6703 | 0.432 (0.418, 0.436) | -0.56 (-0.59, -0.33) |
| 621 | yellow-poplar | *Liriodendron* | *tulipifera* | 0.4368 | -0.06007 | 0.6302 | 0.489 (0.489, 0.492) | 0.799 (0.570, 0.799) |
| 631 | tanoak | *Lithocarpus* | *densiflorus* | 0.3184 | -0.06092 | 0.6164 | 0.365 (0.362, 0.375) | -2.14 (-2.66, -2.14) |
| 641 | Osage-orange | *Maclura* | *pomifera* | 0.483 | -0.0588 | 0.4345 | 0.383 (0.367, 0.662) | -6.98 (-7.96, -0.48) |
| 650 | magnolia_sp. | *Magnolia* | *sp.* | 0.374 | -0.06033 | 0.661 | 0.497 (0.402, 0.573) | -5.02 (-5.95, 1.895) |
| 651 | cucumbertree | *Magnolia* | *acuminata* | 0.3866 | -0.05992 | 0.6499 | 0.519 (0.469, 0.581) | 0.117 (-0.28, 1.770) |
| 652 | southern_magnolia | *Magnolia* | *grandiflora* | 0.3689 | -0.06085 | 0.6003 | 0.868 (0.793, 0.997) | -0.28 (-1.62, 0.347) |
| 653 | sweetbay | *Magnolia* | *virginiana* | 0.4052 | -0.06015 | 0.6132 | 0.454 (0.412, 0.470) | -1.35 (-1.91, -1.14) |
| 654 | bigleaf_magnolia | *Magnolia* | *macrophylla* | 0.3704 | -0.06013 | 0.6668 | 0.970 (0.900, 0.999) | -2.76 (-7.46, -0.16) |
| 655 | mountain_magnolia | *Magnolia* | *fraseri* | 0.4144 | -0.06151 | 0.6318 | 0.559 (0.456, 0.681) | -6.43 (-7.91, -3.72) |
| 660 | apple_sp. | *Malus* | *sp.* | 0.5128 | -0.05854 | 0.3544 | 0.350 (0.324, 0.420) | -4.38 (-7.64, -1.43) |
| 661 | Oregon_crab_apple | *Malus* | *fusca* | 0.4456 | -0.05893 | 0.4488 | 0.415 (0.011, 0.960) | -2.34 (-7.52, 7.046) |
| 680 | mulberry_sp. | *Morus* | *sp.* | 0.4192 | -0.05945 | 0.4543 | 0.571 (0.425, 0.755) | -4.82 (-7.47, -0.47) |
| 681 | white_mulberry | *Morus* | *alba* | 0.4507 | -0.05869 | 0.4644 | 0.712 (0.525, 0.945) | -4.51 (-7.87, 1.751) |
| 682 | red_mulberry | *Morus* | *rubra* | 0.4864 | -0.05908 | 0.4418 | 0.613 (0.498, 0.758) | -1.83 (-7.48, -1.74) |
| 691 | water_tupelo | *Nyssa* | *aquatica* | 0.4224 | -0.06097 | 0.5739 | 0.485 (0.461, 0.516) | -0.13 (-1.55, -0.21) |
| 692 | Ogeechee_tupelo | *Nyssa* | *ogeche* | 0.3737 | -0.0607 | 0.5925 | 0.235 (0.053, 0.977) | 2.435 (-7.01, 7.818) |
| 693 | blackgum | *Nyssa* | *sylvatica* | 0.3566 | -0.06209 | 0.6207 | 0.503 (0.489, 0.521) | -1.98 (-2.72, -1.78) |
| 694 | swamp_tupelo | *Nyssa* | *sylvatica_var*  *biflora* | 0.464 | -0.0602 | 0.5688 | 0.470 (0.449, 0.493) | 0.391 (-0.51, 1.217) |
| 701 | eastern  hophornbeam | *Ostrya* | *virginiana* | 0.4454 | -0.05726 | 0.5438 | 0.557 (0.506, 0.604) | -3.19 (-4.03, -2.39) |
| 711 | sourwood | *Oxydendrum* | *arboreum* | 0.3931 | -0.06144 | 0.5876 | 0.475 (0.456, 0.494) | -2.25 (-2.68, -1.92) |
| 712 | paulownia  or_empress-tree | *Paulownia* | *tomentosa* | 0.4665 | -0.06016 | 0.528 | 0.426 (0.313, 0.780) | -3.95 (-5.81, 1.851) |
| 721 | redbay | *Persea* | *borbonia* | 0.4233 | -0.05827 | 0.5463 | 0.453 (0.382, 0.482) | -0.92 (-1.79, 0.002) |
| 722 | water-elm  or_planertree | *Planera* | *aquatica* | 0.385 | -0.05934 | 0.4696 | 0.820 (0.610, 0.998) | -2.25 (-7.40, 0.625) |
| 730 | California_sycamore | *Platanus* | *racemosa* | 0.4402 | -0.0582 | 0.5467 | - |
| 731 | sycamore | *Platanus* | *occidentalis* | 0.4994 | -0.05722 | 0.5417 | 0.578 (0.564, 0.621) | -0.34 (-1.98, -0.17) |
| 740 | Populus_sp. | *Populus* | *sp.* | 0.4075 | -0.05975 | 0.6047 | 0.675 (0.622, 0.735) | 3.081 (1.569, 4.218) |
| 741 | balsam_poplar | *Populus* | *balsamifera* | 0.4062 | -0.06004 | 0.5705 | 0.345 (0.330, 0.367) | 2.040 (1.296, 2.965) |
| 742 | eastern_cottonwood | *Populus* | *deltoides* | 0.4512 | -0.06039 | 0.5405 | 0.599 (0.533, 0.686) | 3.460 (1.794, 4.328) |
| 743 | bigtooth_aspen | *Populus* | *grandidentata* | 0.4359 | -0.06067 | 0.6066 | 0.424 (0.401, 0.440) | 3.080 (2.554, 3.605) |
| 744 | swamp_cottonwood | *Populus* | *heterophylla* | 0.3953 | -0.06007 | 0.5966 | 0.959 (0.881, 0.998) | -0.43 (-3.82, 1.792) |
| 745 | plains_cottonwood | *Populus* | *deltoides_sp*  *monilifera* | 0.3975 | -0.06066 | 0.5359 | 0.358 (0.034, 0.983) | 5.419 (-7.29, 7.666) |
| 746 | quaking_aspen | *Populus* | *tremuloides* | 0.351 | -0.0604 | 0.6078 | 0.404 (0.394, 0.410) | 1.975 (1.815, 2.215) |
| 747 | black_cottonwood | *Populus* | *trichocarpa* | 0.3491 | -0.05962 | 0.6559 | 0.392 (0.331, 0.482) | 6.923 (3.349, 7.841) |
| 748 | Fremont_or  Rio_Grande  cottonwood | *Populus* | *fremontii* | 0.3783 | -0.06036 | 0.5398 | - |
| 749 | narrowleaf  cottonwood | *Populus* | *angustifolia* | 0.3765 | -0.061 | 0.5156 | 0.383 (0.298, 0.421) | 6.489 (3.634, 7.123) |
| 752 | silver_poplar | *Populus* | *alba* | 0.3969 | -0.05937 | 0.5751 | - |
| 755 | mesquite_sp. | *Prosopis* | *sp.* | 0.35 | -0.128 | 0.4937 | - |
| 756 | western  honey_mesquite | *Prosopis* | *glandulosa_var*  *torreyana* | 0.3856 | -0.1701 | 0.5051 | 0.635 (0.057, 0.984) | -3.45 (-7.39, 7.541) |
| 757 | velvet_mesquite | *Prosopis* | *velutina* | 0.3853 | -0.1444 | 0.4957 | 0.885 (0.057, 0.982) | -3.09 (-7.92, 6.633) |
| 758 | screwbean_mesquite | *Prosopis* | *pubescens* | 0.3858 | -0.1288 | 0.5115 | - |
| 760 | cherry_and_plum_sp. | *Prunus* | *sp.* | 0.3486 | -0.05871 | 0.6071 | 0.758 (0.194, 0.941) | -4.01 (-7.93, 6.164) |
| 761 | pin_cherry | *Prunus* | *pensylvanica* | 0.4275 | -0.05803 | 0.5369 | 0.482 (0.434, 0.546) | -0.83 (-0.79, -0.20) |
| 762 | black_cherry | *Prunus* | *serotina* | 0.4527 | -0.05745 | 0.525 | 0.520 (0.516, 0.540) | -0.96 (-1.31, -0.88) |
| 763 | chokecherry | *Prunus* | *virginiana* | 0.3539 | -0.05971 | 0.5676 | 0.803 (0.362, 0.854) | -1.56 (-4.45, -0.03) |
| 765 | Canada_plum | *Prunus* | *nigra* | 0.3846 | -0.05842 | 0.5536 | - |
| 766 | wild_plum | *Prunus* | *americana* | 0.3602 | -0.0588 | 0.4735 | 0.772 (0.014, 0.921) | 4.515 (-7.82, 7.694) |
| 768 | bitter_cherry | *Prunus* | *emarginata* | 0.3915 | -0.05859 | 0.6802 | 0.277 (0.262, 0.353) | -1.07 (-2.15, 0.519) |
| 800 | oak_sp._deciduous | *Quercus* | *sp.* | 0.3693 | -0.0696 | 0.5332 | 0.745 (0.642, 0.991) | -5.70 (-7.61, 1.046) |
| 801 | coast_live_oak | *Quercus* | *agrifolia* | 0.3638 | -0.06946 | 0.579 | 0.461 (0.453, 0.481) | -1.73 (-1.91, -0.68) |
| 802 | white_oak | *Quercus* | *alba* | 0.3989 | -0.06923 | 0.6029 | 0.543 (0.535, 0.552) | -1.21 (-1.43, -1.08) |
| 803 | Arizona_white_oak | *Quercus* | *arizonica* | 0.3623 | -0.097 | 0.5553 | 0.477 (0.053, 0.986) | -6.59 (-7.30, 7.613) |
| 804 | swamp_white_oak | *Quercus* | *bicolor* | 0.4448 | -0.06943 | 0.5482 | 0.518 (0.443, 0.670) | -4.30 (-4.64, -0.10) |
| 805 | canyon_live_oak | *Quercus* | *chrysolepis* | 0.2034 | -0.06893 | 0.5863 | 0.349 (0.326, 0.352) | -1.00 (-1.13, -0.66) |
| 806 | scarlet_oak | *Quercus* | *coccinea* | 0.4052 | -0.06901 | 0.5969 | 0.576 (0.557, 0.581) | -0.62 (-0.99, -0.41) |
| 807 | blue_oak | *Quercus* | *douglasii* | 0.3635 | -0.06902 | 0.5787 | 0.453 (0.440, 0.474) | 4.196 (4.240, 5.133) |
| 808 | Durand_oak | *Quercus* | *durandii* | 0.3632 | -0.06839 | 0.6078 | 0.780 (0.571, 0.959) | -1.39 (-4.02, 7.748) |
| 809 | northern_pin_oak | *Quercus* | *ellipsoidalis* | 0.4155 | -0.06988 | 0.5438 | 0.508 (0.445, 0.563) | -2.18 (-3.46, -0.62) |
| 810 | Emory_oak | *Quercus* | *emoryi* | 0.3639 | -0.1332 | 0.5924 | 0.235 (0.009, 0.959) | -5.59 (-7.53, 7.574) |
| 811 | Engelmann_oak | *Quercus* | *engelmannii* | 0.364 | -0.0695 | 0.5794 | - |
| 812 | southern_red_oak | *Quercus* | *falcata_var*  *falcata* | 0.3558 | -0.06998 | 0.631 | 0.639 (0.621, 0.642) | 0.323 (-0.14, 0.577) |
| 813 | cherrybark_oak | *Quercus* | *falcata_var*  *pagodifolia* | 0.3724 | -0.06897 | 0.6107 | 0.651 (0.544, 0.705) | 2.312 (0.051, 3.246) |
| 814 | Gambel_oak | *Quercus* | *gambelii* | 0.3632 | -0.09878 | 0.7387 | 0.359 (0.022, 0.964) | -5.98 (-7.78, 7.789) |
| 815 | Oregon_white_oak | *Quercus* | *garryana* | 0.1322 | -0.06879 | 0.6329 | 0.379 (0.362, 0.406) | 2.863 (2.527, 3.657) |
| 816 | bear_oak  or_scrub_oak | *Quercus* | *ilicifolia* | 0.3216 | -0.06851 | 0.5612 | 0.399 (0.304, 0.997) | -5.20 (-6.77, 0.983) |
| 817 | shingle_oak | *Quercus* | *imbricaria* | 0.388 | -0.06963 | 0.5743 | 0.581 (0.525, 0.636) | -0.51 (-1.94, -0.01) |
| 818 | California_black_oak | *Quercus* | *kelloggii* | 0.3228 | -0.06908 | 0.5092 | 0.457 (0.433, 0.466) | 2.533 (2.081, 2.743) |
| 819 | turkey_oak | *Quercus* | *laevis* | 0.3134 | -0.06855 | 0.5731 | 0.344 (0.320, 0.441) | -0.84 (-1.60, 1.683) |
| 820 | laurel_oak | *Quercus* | *laurifolia* | 0.4013 | -0.06834 | 0.5799 | 0.634 (0.583, 0.638) | -0.41 (-1.33, -0.14) |
| 821 | California_white_oak | *Quercus* | *lobata* | 0.369 | -0.06962 | 0.5842 | 0.455 (0.441, 0.532) | -4.29 (-5.49, -2.75) |
| 822 | overcup_oak | *Quercus* | *lyrata* | 0.3783 | -0.06895 | 0.5892 | 0.428 (0.391, 0.491) | -3.27 (-6.33, -1.22) |
| 823 | bur_oak | *Quercus* | *macrocarpa* | 0.3424 | -0.06953 | 0.5546 | 0.489 (0.467, 0.492) | -1.68 (-2.70, -1.68) |
| 824 | blackjack_oak | *Quercus* | *marilandica* | 0.3371 | -0.06795 | 0.5355 | 0.380 (0.362, 0.477) | -3.01 (-3.13, -1.12) |
| 825 | swamp_chestnut_oak | *Quercus* | *michauxii* | 0.3891 | -0.07133 | 0.6183 | 0.473 (0.461, 0.548) | -2.13 (-4.77, -1.52) |
| 826 | chinkapin_oak | *Quercus* | *muehlenbergii* | 0.3833 | -0.06995 | 0.5787 | 0.714 (0.687, 0.802) | -1.76 (-3.17, -0.86) |
| 827 | water_oak | *Quercus* | *nigra* | 0.4053 | -0.06958 | 0.6223 | 0.590 (0.565, 0.592) | -1.07 (-1.40, -0.66) |
| 828 | Nuttall_oak | *Quercus* | *nuttallii* | 0.4071 | -0.06886 | 0.5985 | 0.038 (0.046, 0.894) | -2.50 (-6.06, 5.346) |
| 829 | Mexican_blue_oak | *Quercus* | *oblongifolia* | 0.3637 | -0.07205 | 0.5392 | 0.777 (0.044, 0.981) | 5.993 (-7.25, 7.325) |
| 830 | pin_oak | *Quercus* | *palustris* | 0.3969 | -0.06978 | 0.6021 | 0.450 (0.417, 0.495) | -0.87 (-2.96, -0.70) |
| 831 | willow_oak | *Quercus* | *phellos* | 0.3963 | -0.06991 | 0.5934 | 0.579 (0.558, 0.598) | -0.53 (-1.44, -0.25) |
| 832 | chestnut_oak | *Quercus* | *prinus* | 0.4206 | -0.06931 | 0.5805 | 0.509 (0.502, 0.517) | -1.83 (-1.93, -1.40) |
| 833 | northern_red_oak | *Quercus* | *rubra* | 0.4363 | -0.06878 | 0.5678 | 0.538 (0.537, 0.548) | -1.24 (-1.29, -0.89) |
| 834 | Shumard_oak | *Quercus* | *shumardii* | 0.4463 | -0.06835 | 0.5622 | 0.964 (0.869, 0.997) | 0.253 (-0.67, 2.067) |
| 835 | post_oak | *Quercus* | *stellata* | 0.3291 | -0.07089 | 0.6147 | 0.526 (0.498, 0.529) | -1.02 (-1.88, -0.96) |
| 836 | Delta_post_oak | *Quercus* | *stellata_var*  *mississippiensis* | 0.3746 | -0.06882 | 0.6043 | 0.312 (0.038, 0.949) | -5.10 (-7.37, 7.588) |
| 837 | black_oak | *Quercus* | *velutina* | 0.3725 | -0.06928 | 0.6039 | 0.507 (0.503, 0.517) | -1.60 (-1.60, -1.15) |
| 838 | live_oak | *Quercus* | *virginiana* | 0.3613 | -0.06859 | 0.5469 | 0.639 (0.633, 0.669) | -1.13 (-2.77, -0.93) |
| 839 | interior_live_oak | *Quercus* | *wislizeni* | 0.3641 | -0.07064 | 0.5801 | 0.533 (0.493, 0.600) | 2.755 (2.296, 3.167) |
| 840 | dwarf_post_oak | *Quercus* | *stellata_var*  *margaretta* | 0.2764 | -0.06933 | 0.6379 | 0.422 (0.242, 0.602) | 3.874 (-0.46, 4.695) |
| 841 | dwarf_live_oak | *Quercus* | *minima* | 0.3101 | -0.07011 | 0.5181 | 0.010 (0.012, 0.970) | 2.071 (-7.90, 7.597) |
| 842 | bluejack_oak | *Quercus* | *incana* | 0.3064 | -0.06852 | 0.556 | 0.522 (0.322, 0.822) | -0.22 (-6.09, 1.656) |
| 843 | silverleaf_oak | *Quercus* | *hypoleucoildes* | 0.3639 | 0.06204 | 0.4539 | 0.516 (0.066, 0.935) | -6.98 (-6.81, 7.606) |
| 844 | Oglethorpe_oak | *Quercus* | *oglethorpensis* | 0.3638 | -0.06979 | 0.5791 | - |
| 845 | dwarf_chinkapin_oak | *Quercus* | *prinoides* | 0.3634 | -0.06943 | 0.5785 | - |
| 846 | gray_oak | *Quercus* | *grisea* | 0.3635 | -0.07015 | 0.5791 | - |
| 850 | oak_evergreen | *Quercus* | *sp.* | 0.3629 | -0.09528 | 0.53 | 0.745 (0.642, 0.991) | -5.70 (-7.61, 1.046) |
| 899 | scrub_oak | *Quercus* | *sp.* | 0.3638 | -0.07045 | 0.5791 | 0.419 (0.030, 0.828) | -5.60 (-7.40, 7.529) |
| 901 | black_locust | *Robinia* | *pseudoacacia* | 0.4268 | -0.05959 | 0.5418 | 0.512 (0.476, 0.526) | -0.37 (-0.67, -0.03) |
| 902 | New_Mexico_locust | *Robinia* | *neomexicana* | 0.4136 | -0.05992 | 0.5466 | - |
| 911 | palmetto_sp. | *Sabal* | *sp.* | 0.4364 | -0.05797 | 0.5914 | 0.907 (0.161, 0.985) | -0.62 (-3.17, 6.712) |
| 919 | western_soapberry | *Sapindus* | *drummondii* | 0.3905 | -0.06025 | 0.5266 | - |
| 920 | willow_sp. | *Salix* | *sp.* | 0.4642 | -0.06033 | 0.507 | 0.224 (0.224, 0.441) | 2.521 (1.955, 6.367) |
| 921 | peachleaf_willow | *Salix* | *amygdaloides* | 0.3631 | -0.06065 | 0.4677 | 0.639 (0.009, 0.955) | -2.74 (-7.56, 7.726) |
| 922 | black_willow | *Salix* | *nigra* | 0.4914 | -0.06072 | 0.4416 | 0.523 (0.497, 0.647) | -1.50 (-0.97, 0.832) |
| 923 | diamond_willow | *Salix* | *eriocephala* | 0.4304 | -0.05882 | 0.4867 | - |
| 927 | white_willow | *Salix* | *alba* | 0.4421 | -0.06086 | 0.4719 | 0.254 (0.018, 0.971) | 7.720 (-7.05, 7.677) |
| 931 | sassafras | *Sassafras* | *albidum* | 0.4113 | -0.05929 | 0.5707 | 0.450 (0.421, 0.475) | -1.26 (-2.02, -0.99) |
| 935 | American  mountain_ash | *Sorbus* | *americana* | 0.4363 | -0.06021 | 0.486 | 0.556 (0.408, 0.978) | -1.39 (-6.50, 0.829) |
| 936 | European  mountain_ash | *Sorbus* | *aucuparia* | 0.4172 | -0.06018 | 0.5175 | - |
| 950 | basswood_sp. | *Tilia* | *sp.* | 0.3827 | -0.05938 | 0.5871 | 0.431 (0.409, 0.500) | -3.43 (-3.59, -1.30) |
| 951 | American_basswood | *Tilia* | *americana* | 0.4258 | -0.05928 | 0.5768 | 0.462 (0.449, 0.475) | -1.28 (-1.45, -0.93) |
| 952 | white_basswood | *Tilia* | *heterophylla* | 0.4142 | -0.05891 | 0.6273 | 0.880 (0.071, 0.976) | 6.009 (-7.72, 7.293) |
| 953 | Carolina_basswood | *Tilia* | *americana_var*  *caroliniana* | 0.4413 | -0.06022 | 0.596 | - |
| 970 | elm_sp. | *Ulmus* | *sp.* | 0.3526 | -0.05875 | 0.5631 | 0.286 (0.286, 0.581) | -7.64 (-6.68, 1.082) |
| 971 | winged_elm | *Ulmus* | *alata* | 0.3401 | -0.05744 | 0.6205 | 0.717 (0.645, 0.749) | -0.70 (-1.84, -0.49) |
| 972 | American_elm | *Ulmus* | *americana* | 0.4192 | -0.05775 | 0.5523 | 0.536 (0.534, 0.571) | -1.75 (-1.81, -1.15) |
| 973 | cedar_elm | *Ulmus* | *crassifolia* | 0.37 | -0.0577 | 0.5649 | 0.135 (0.030, 0.879) | 3.652 (-7.10, 7.717) |
| 974 | Siberian_elm | *Ulmus* | *pumila* | 0.3404 | -0.0571 | 0.5674 | 0.457 (0.293, 0.601) | -1.30 (-7.49, 2.512) |
| 975 | slippery_elm | *Ulmus* | *rubra* | 0.394 | -0.05768 | 0.6015 | 0.603 (0.539, 0.640) | -1.02 (-2.44, -0.98) |
| 976 | September_elm | *Ulmus* | *serotina* | 0.4359 | -0.05864 | 0.6239 | - |
| 977 | rock_elm | *Ulmus* | *thomasii* | 0.3863 | -0.05777 | 0.6185 | 0.571 (0.413, 0.947) | 0.815 (-7.60, 4.719) |
| 981 | California-laurel | *Umbellularia* | *californica* | 0.481 | -0.05967 | 0.4939 | 0.605 (0.581, 0.675) | 4.048 (3.637, 4.440) |
| 989 | mangrove | *Rhizophora* | *mangle* | 0.4058 | -0.06001 | 0.5514 | - |
| 990 | tesota_or  Arizona-ironwood | *Olneya* | *tesota* | 0.4063 | -0.05628 | 0.5511 | - |
| 991 | saltcedar | *Tamarix* | *sp.* | 0.406 | -0.06027 | 0.5518 | - |
| 992 | melaleuca | *Melaleuca* | *quinquenervia* | 0.3984 | -0.05969 | 0.5239 | 0.479 (0.008, 0.971) | -1.71 (-7.64, 7.136) |
| 993 | chinaberry | *Melia* | *azedarach* | 0.4526 | -0.05833 | 0.5408 | 0.418 (0.369, 0.804) | -5.37 (-6.63, 1.340) |
| 994 | Chinese_tallowtree | *Sapium* | *sebiferum* | 0.3878 | -0.05995 | 0.5945 | 0.336 (0.223, 0.457) | 1.951 (-1.30, 2.776) |
| 995 | tung-oil-tree | *Aleurites* | *fordii* | 0.3362 | -0.06159 | 0.6067 | - |
| 996 | smoketree | *Cotinus* | *obovatus* | 0.3649 | -0.05874 | 0.4767 | 0.162 (0.006, 0.968) | 3.363 (-7.17, 7.051) |
| 997 | Russian-olive | *Elaeagnus* | *angustifolia* | 0.3203 | -0.05848 | 0.4891 | - |
